# Chronic morbidity, deprivation and primary medical care spending in England in 2015-16: a cross-sectional spatial analysis

**DOI:** 10.1186/s12916-017-0996-0

**Published:** 2018-02-14

**Authors:** Evangelos Kontopantelis, Mamas A. Mamas, Harm van Marwijk, Andrew M. Ryan, Peter Bower, Bruce Guthrie, Tim Doran

**Affiliations:** 1 Division of Population Health, Health Services Research & Primary Care; Faculty of Biology, Medicine and Health, University of Manchester, Greater Manchester, UK; 2 NIHR School for Primary Care Research, Faculty of Biology, Medicine and Health, University of Manchester, 5th Floor Williamson Building, Greater Manchester, UK; 3 Science and Technology in Medicine, Keele University, Staffordshire, UK; 4 School of Public Health, University of Michigan, Ann Arbor, MI USA; 5 Population Health Sciences Division, School of Medicine, University of Dundee, Dundee, UK; 6 Department of Health Sciences, University of York, Yorkshire, UK

**Keywords:** Primary care funding, Chronic conditions, Morbidity, Deprivation, Spatial clustering, Quality and Outcomes Framework, QOF, England, UK, Carr–Hill formula, Global sum allocation formula

## Abstract

**Background:**

Primary care provides the foundation for most modern health-care systems, and in the interests of equity, it should be resourced according to local need. We aimed to describe spatially the burden of chronic conditions and primary medical care funding in England at a low geographical level, and to measure how much variation in funding is explained by chronic condition prevalence and other patient and regional factors.

**Methods:**

We used multiple administrative data sets including chronic condition prevalence and management data (2014/15), funding for primary-care practices (2015-16), and geographical and area deprivation data (2015). Data were assigned to a low geographical level (average 1500 residents). We investigated the overall morbidity burden across 19 chronic conditions and its regional variation, spatial clustering and association with funding and area deprivation. A linear regression model was used to explain local variation in spending using patient demographics, morbidity, deprivation and regional characteristics.

**Results:**

Levels of morbidity varied within and between regions, with several clusters of very high morbidity identified. At the regional level, morbidity was modestly associated with practice funding, with the North East and North West appearing underfunded. The regression model explained 39% of the variability in practice funding, but even after adjusting for covariates, a large amount of variability in funding existed across regions. High morbidity and, especially, rural location were very strongly associated with higher practice funding, while associations were more modest for high deprivation and older age.

**Conclusions:**

Primary care funding in England does not adequately reflect the contemporary morbidity burden. More equitable resource allocation could be achieved by making better use of routinely available information and big data resources. Similar methods could be deployed in other countries where comparable data are collected, to identify morbidity clusters and to target funding to areas of greater need.

**Electronic supplementary material:**

The online version of this article (10.1186/s12916-017-0996-0) contains supplementary material, which is available to authorized users.

## Background

Primary care plays a vital role in coordinating care and managing demand in the community, and provides the cornerstone of many modern health-care systems [1]. Systems with a strong primary- care focus are associated with lower care costs, higher patient satisfaction levels, better overall health, lower medication use and a decrease in hospitalisation and emergency department visits [2, 3]. Primary-care-focused health care can also reduce the impact of socioeconomic factors on health [3], leading to improvements in overall health and reductions in health inequalities across population subgroups [4].

One of the ongoing challenges for primary care is to tackle variability in outcomes, and to contribute towards breaking the link between wealth and health [5]. Whilst coordinated primary-care initiatives can help to reduce health inequalities, they can also unintentionally increase them: universal interventions that are effective in improving population health, such as smoking cessation programmes [6], often increase inequities because they are more effective in affluent areas [7]. Efforts to improve equity are also undermined if resources are not allocated according to need. The UK’s National Health Service (NHS) is built on a strong primary-care base and provides universal comprehensive care free at the point of delivery, but striking inequities in health outcomes remain [8].

In an attempt to address this, in the early 2000s the UK government pursued a range of interventions intended to improve equity and reformed the method of funding primary-care practices. A new national General Medical Services contract for general practitioners was introduced in 2004, with two main funding components. Core funding for essential and key additional services was calculated using the Carr–Hill global sum formula [9, 10], based on the number of registered patients, adjusted for patient factors (including age, sex, turnover, morbidity and mortality) and the local context (staff market forces and rurality). This core funding was supplemented by a new pay-for-performance programme – the Quality and Outcomes Framework (QOF) – introduced with the aim of increasing overall funding for primary care and reducing variation in quality between providers [11]. QOF payments were dependent on practice performance against over 100 quality targets relating to practice organisation, patient experience and clinical management of chronic conditions. Payments for clinical targets were adjusted according to the relative prevalence of the relevant condition in the practice population. Further investment in primary care was targeted at deprived areas in 2007 and 2008 [12], with the aim of supporting local efforts to tackle chronic disease and increasing physician numbers in areas where physician recruitment and health-care delivery can be challenging [13].

For several reasons, these initiatives did not fully address the inequitable distribution of resources and its contribution towards the perpetuation of health inequalities [14]. First, funding under the QOF programme initially favoured larger practices in more affluent areas, which tended to perform better against the quality targets [15, 16]. Gaps in performance rapidly narrowed [15, 17], although there was no clear evidence for improvement in patient outcomes [18], or impact on mortality over the longer term [5, 19]. Second, payment adjustments intended to protect practice incomes had unintended consequences. For the clinical QOF targets, relative prevalence was calculated based on the square root of disease prevalence rather than prevalence itself, and practices with low disease prevalence – below the 5th percentile – were treated as if prevalence were equal to the 5th percentile. These adjustments were intended to retain parity between practices with respect to quality payments, but had the effect of uncoupling the relationship between workload and remuneration, disadvantaging practices with high disease prevalence, which were more likely to be in deprived areas. For this reason, from 2009 onwards the prevalence adjustment was calculated based on actual prevalence. Third, the global sum formula for core funding does not directly adjust for patient deprivation and uses a measure of morbidity based on Standardised Limited Long-Standing Illness data derived from the 1998–2000 Health Survey for England [9]. There are long-standing concerns that this formula does not fully reflect the pressures and costs that deprivation imposes on practices [20]. In response to these concerns, under the 2004 contract, practices received a Minimum Practice Income Guarantee (MPIG), a correction payment to prevent their core funding, based on the new global sum formula, falling below historical levels. MPIG began to be phased out in 2014 with aim of equalising weighted funding per patient across all practices by 2021 [20, 21]. This has left many practices in deprived areas facing financial hardship and urgently calling for a fairer system of resource allocation [21]. NHS England, NHS Employers and the British Medical Association are committed to revising the Carr–Hill formula to reflect deprivation better [10, 22], but agreement on a new system of allocation has yet to be reached.

Since the introduction of the QOF in 2004, annually updated prevalence data have been available for numerous chronic conditions at the practice level, and these could provide more precise, timely and comprehensive information for determining health-care need. In this study, we aimed to describe the overall chronic condition burden, as measured by prevalence data derived from the 2014/15 QOF, and to evaluate its association with primary medical care funding in England for 2015-16. More specifically, we aimed to (a) spatially describe the overall chronic condition burden and payments to primary-care practices at a low geographical level; (b) quantify and describe the variability and spatial clustering in these two measures, across ten English regions and (c) measure how much of the variation in spending is explained by the overall chronic condition burden and how much by other relevant population factors measurable at a low geographical level, in particular area deprivation.

## Methods

Our primary unit of analysis was the Lower Layer Super Output Area (LSOA) in England: 32,844 geographical administrative units with an average population of 1500. Deprivation was measured though the 2015 Index of Multiple Deprivation (IMD) [23]. To measure the morbidity burden we created a chronic morbidity index (CMI), calculated as the sum of 19 chronic condition registers in the 2014/15 QOF, divided by the total practice population. Unfortunately, the measure cannot capture comorbidity since that information is not recorded within the QOF. Someone with two conditions would be entered independently in the two respective registers. NHS payments to general practices for 2015-16 were reported by NHS Digital, covering all centrally managed payment schemes (global sum, MPIG, balance of PMS (Personal Medical Services) expenditure, QOF and enhanced services) and also the decentralised Local Enhanced Services scheme. Although other local payments were not captured (for example, Local Authority public health funding) the reported payments were in effect the bulk of the income for general practices [24]. Both funding and the CMI were assigned to LSOAs using methodology previously described [12]. Further details of the data and the methods used to attribute them to the LSOA level are provided in Additional file 1: Appendix 1.

### Analyses

The outcome of interest was average primary medical funding per patient in 2015-16. The key covariates were the IMD 2015 and the CMI of the 2014/15 QOF measured at the LSOA level. Correlations between the IMD and the CMI were calculated using Pearson’s rho for the whole of England and each of the ten regions (at the LSOA level, weighted for 2014 population estimates). Funding and the CMI were visualised using spatial maps for all of England and each region. After aggregating at the regional level (weighting for 2014 LSOA populations), scatter plots were used to describe the relationship between the outcome and each of the two key covariates. Box plots were plotted to describe the distribution of funding and CMI within each region.

Spatial autocorrelation for funding per person, deprivation and morbidity (correlation in a signal among nearby locations in space) was assessed and quantified using Moran’s *I*. This measure accounts for the multi-dimensional and multi-directional nature of spatial autocorrelation, and can identify the presence of clusters. A higher value than the one expected under a random spatial pattern would indicate that, using morbidity as an example, areas with high levels of morbidity are clustered together and, hence, a high morbidity LSOA is more likely to be bordered by (or have as close neighbours) LSOAs with similarly high morbidity levels. We calculated Moran’s *I* for each region and the whole of England, to allow for within-England comparisons.

A linear regression model, weighted for 2015 LSOA size, was used to quantify the strength of association between average primary medical care funding per person and region, demographic characteristics (age, sex and ethnicity), urbanity, the CMI and the IMD. A second model with interaction terms is discussed in Additional file 1: Appendix 1. Our choice between unadjusted (denominator = attribution population) and adjusted (denominator = LSOA population) per patient cost was informed by the predictive power of the regression models, with the former metric leading to better models. Hence, all analyses and graphs we present use unadjusted funding.

Analyses were executed with Stata v14.1 and R v3.3.1. Most comparisons are statistically significant due to the size of the data set and thus, we focus on effects sizes where possible. All variables we used were complete.

## Results

**Fig. 1:**
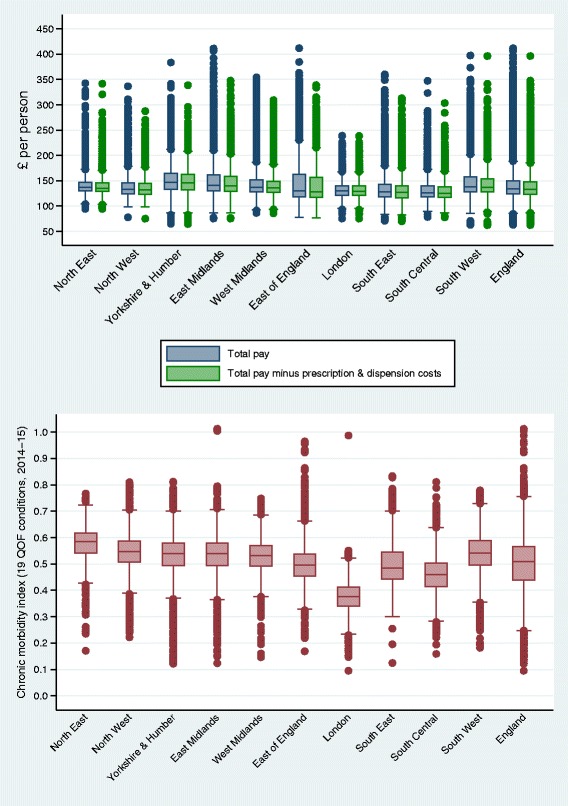
Box plots of average primary medical care spending for 2015-16 (top) and the chronic morbidity index for 2014/15 (bottom), across English regions, weighted for LSOA population sizes. LSOA Lower Layer Super Output Area, QOF Quality and Outcomes Framework

A total of 56,924,424 patients were registered with a general practice in January 2015. Total funding for practices in 2015-16 was £7.61 billion. Based on the number of registered patients, the median annual primary medical care funding per patient, minus prescription and dispensing costs, was £133.7 at the LSOA level [interquartile range (IQR), £123.5 to £148.0] (Table 1 and Fig. 1). Total median regional spending per person varied from £125.4 in South Central to £145.7 in Yorkshire and the Humber. Based on population estimates rather than registers, funding per person was higher, at £140.4 (IQR, £128.4 to £156.9). The median CMI for England was 0.51 conditions at the LSOA level (IQR, 0.45 to 0.57), with the highest levels observed in the North East (median, 0.59; IQR, 0.54 to 0.62) and the lowest in London (median, 0.38; IQR, 0.34; 75th centile, 0.42). Correlations between the IMD and the CMI, weighted for LSOA population sizes in 2014 (rather than 2015, since the CMI is based on 2014/15 QOF data), were very weak for each region and close to zero for England. The largest (absolute) rho was observed for the West Midlands (-0.194), and the rho for the whole of England was 0.004.

**Table 1:** Characteristics at a low geographical area (LSOA) across England and each of its ten regions*

	England	North East	North West	Yorkshire and Humber	East Midlands	West Midlands	East of England	London	South East	South Central	South West
Aggregates across LSOAs											
Number of LSOAs	32844	1657	4497	3317	2774	3487	3614	4835	2773	2609	3281
Total population	54316618	2618710	7132991	5360027	4637413	5713284	6018383	8538689	4594865	4278953	5423303
Percentage rural	17.0	17.6	9.8	16.5	25.4	14.8	28.3	0.2	20.1	19.8	30.2
Medians (25th and 75th centiles) across LSOAs											
CMI**	0.51 (0.45,0.57)	0.59 (0.54,0.62)	0.55 (0.51,0.59)	0.54 (0.50,0.58)	0.54 (0.50,0.58)	0.53 (0.50,0.57)	0.50 (0.46,0.54)	0.38 (0.34,0.42)	0.49 (0.44,0.55)	0.46 (0.42,0.51)	0.54 (0.50,0.59)
Per patient cost A	134.6 (124.3,150.0)	136.7 (129.9,146.8)	133.6 (124.2,146.0)	146.8 (133.3,164.6)	141.2 (130.2,161.1)	137.1 (127.8,151.3)	129.7 (118.1,162.2)	129.8 (121.7,140.0)	128.1 (118.0,142.5)	126.3 (118.1,140.3)	138.2 (128.7,156.8)
Per patient cost B^†^	133.7 (123.5,148.0)	135.5 (129.0,145.8)	132.7 (123.6,145.0)	145.7 (132.5,162.8)	140.1 (129.4,158.4)	136.1 (127.0,149.0)	128.3 (117.3,156.2)	129.4 (121.3,139.6)	126.8 (117.0,140.3)	125.4 (117.4,138.4)	137.1 (128.1,152.9)
Per patient cost C^‡^	126.6 (118.0,137.1)	130.3 (124.1,139.1)	127.6 (119.1,139.0)	137.3 (126.0,150.0)	131.3 (123.3,139.7)	128.8 (121.4,139.0)	119.7 (111.0,130.7)	126.1 (117.8,136.3)	118.9 (110.7,129.0)	119.0 (112.2,126.9)	128.0 (121.4,135.6)
Adjusted per patient cost A^§^	141.4 (129.3,159.0)	142.7 (134.0,154.2)	140.0 (129.1,154.5)	154.1 (138.7,173.3)	148.5 (136.3,170.0)	143.9 (133.2,160.5)	138.2 (124.0,168.4)	139.0 (128.3,153.5)	133.1 (121.2,147.7)	131.7 (122.2,146.5)	143.5 (132.4,163.0)
Adjusted per patient cost B^†§^	140.4 (128.4,156.9)	141.5 (133.1,152.8)	139.1 (128.3,153.4)	152.8 (138.0,171.3)	147.1 (135.4,165.8)	142.7 (132.4,158.7)	136.3 (122.9,162.8)	138.5 (127.7,153.0)	131.8 (120.2,145.6)	130.7 (121.3,144.5)	142.2 (131.5,158.8)
Adjusted per patient cost C^‡§^	132.4 (122.1,144.7)	135.8 (127.8,145.3)	133.5 (123.3,146.6)	142.5 (130.0,157.8)	136.7 (127.8,147.2)	134.9 (126.3,146.8)	125.6 (115.4,137.6)	135.0 (124.1,149.6)	123.1 (113.7,133.3)	123.5 (115.7,133.4)	132.2 (123.5,141.1)
IMD^¶^	17.4 (9.7,30.1)	23.2 (11.7,38.2)	21.6 (11.0,39.5)	19.8 (11.0,36.9)	16.4 (9.3,29.0)	20.2 (11.4,36.0)	14.5 (8.4,23.0)	22.1 (13.3,32.7)	12.7 (7.3,20.9)	10.7 (5.7,19.3)	15.2 (9.3,23.4)
Percentage female	51.0 (49.8,52.1)	51.3 (50.1,52.4)	51.0 (49.9,52.2)	51.1 (49.9,52.2)	50.9 (49.9,52.0)	50.8 (49.8,51.9)	51.0 (50.0,52.0)	50.8 (49.2,52.1)	51.2 (50.1,52.3)	50.8 (49.7,51.9)	51.2 (50.0,52.3)
Percentage aged 30–59	39.9 (37.4,42.5)	39.4 (37.1,41.6)	39.4 (37.2,41.8)	39.4 (37.0,41.8)	39.5 (37.2,41.8)	38.7 (36.6,40.9)	39.8 (37.4,42.3)	43.0 (40.4,46.3)	39.9 (37.2,42.6)	40.7 (38.0,43.4)	38.5 (36.1,40.9)
Percentage aged 60 or over	23.1 (16.6,29.8)	24.8 (19.8,30.7)	23.5 (18.0,29.8)	23.9 (17.9,29.9)	25.3 (18.4,31.1)	23.7 (17.7,30.4)	24.7 (18.4,31.1)	14.8 (11.3,19.5)	25.2 (19.4,31.1)	23.1 (16.2,29.4)	27.8 (20.3,34.2)
Percentage white British	91.0 (75.3,95.6)	96.8 (94.1,97.9)	94.1 (87.8,96.4)	94.4 (86.1,96.5)	93.7 (83.3,96.2)	89.7 (74.3,95.2)	90.2 (82.1,94.7)	44.4 (28.8,62.0)	90.2 (83.6,93.7)	89.2 (79.1,93.7)	94.8 (91.0,96.5)

*IMD* Index of Multiple Deprivation, *LSOA* Lower Layer Super Output Area

* Variables, in general, reported for the 2015 calendar year, except morbidity (chronic morbidity index, fiscal year 2014/15), costs (fiscal year 2015-16), ethnicity (2011 census)

** Chronic morbidity index, 2014/15

† Minus prescription and dispensing costs

‡ Minus prescription and dispensing costs and drug reimbursement

§ Adjusted for under- and, especially, over-registration using 2015 LSOA population estimates

¶ Index of Multiple Deprivation, details available in the 2015 technical report of the English Indices of Deprivation [23]

**Fig. 2:**
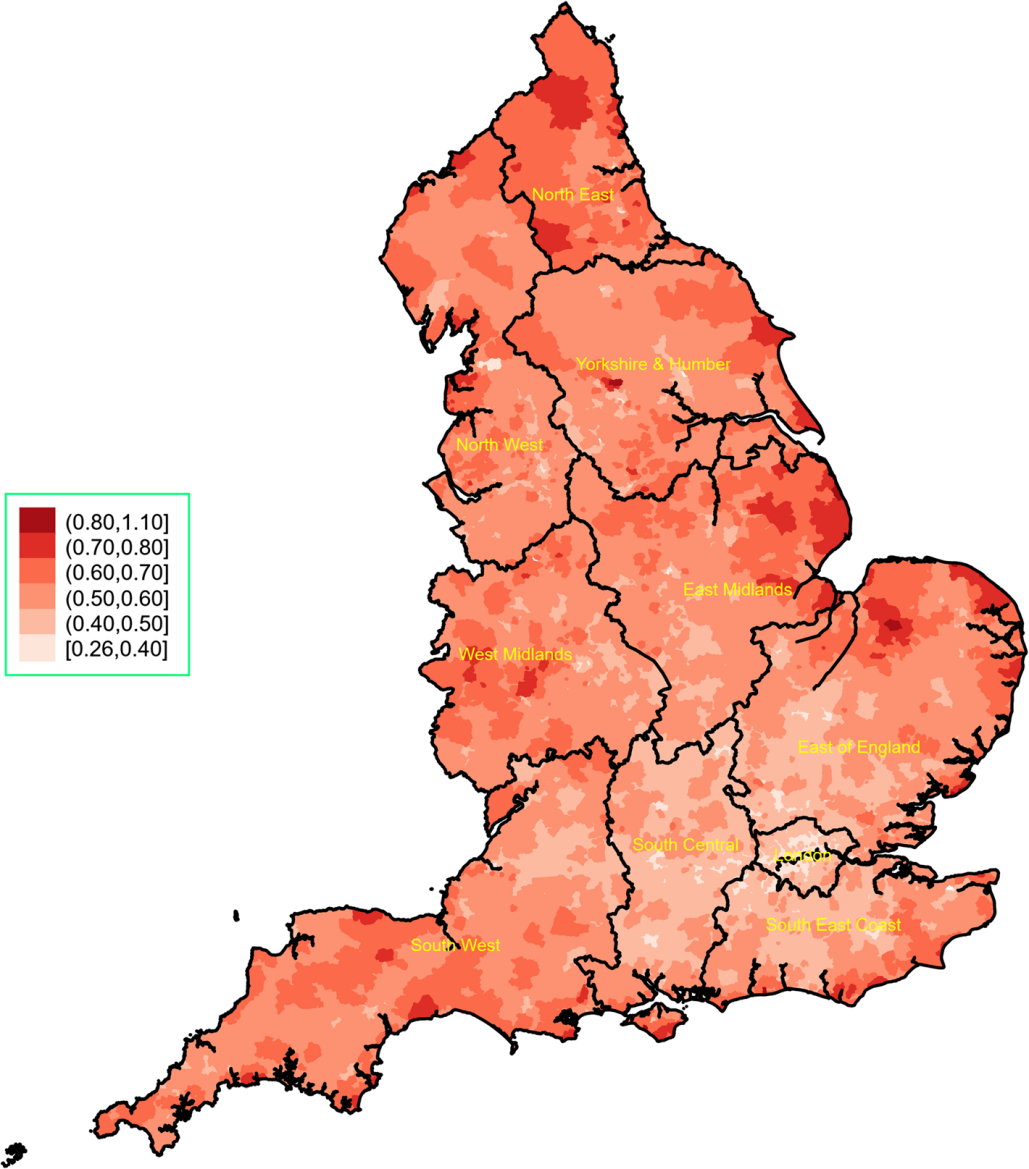
Chronic morbidity index for England, 2014/15. It is calculated as a ratio of the sum of denominators from 19 chronic conditions from the 2014/15 Quality and Outcomes Framework, transposed to the LSOA level, over the LSOA population estimate in 2014. LSOA Lower Layer Super Output Area

**Fig. 3:**
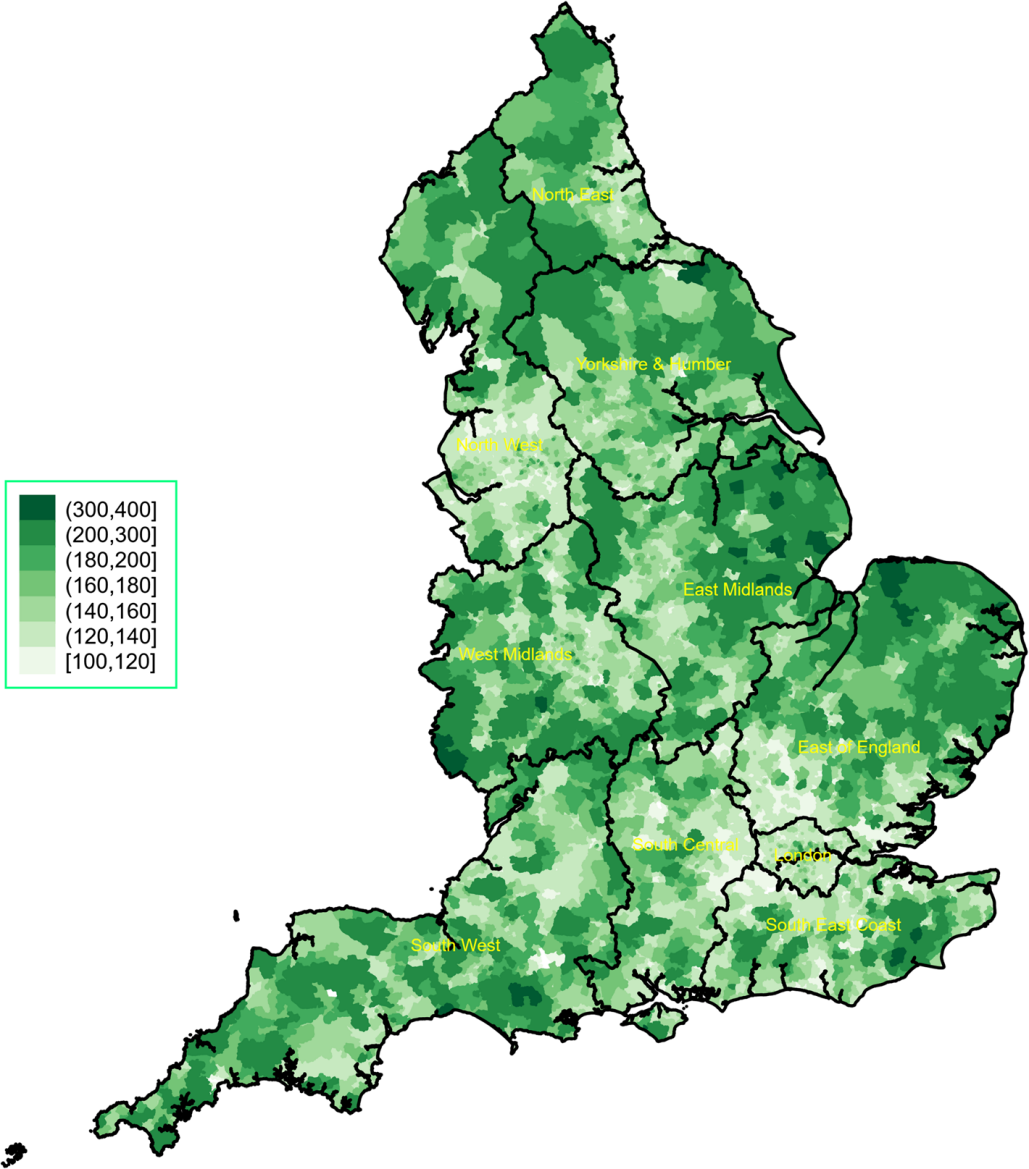
Average primary medical care spending per patient, minus prescription and dispensing costs, 2015-16

**Fig. 4:**
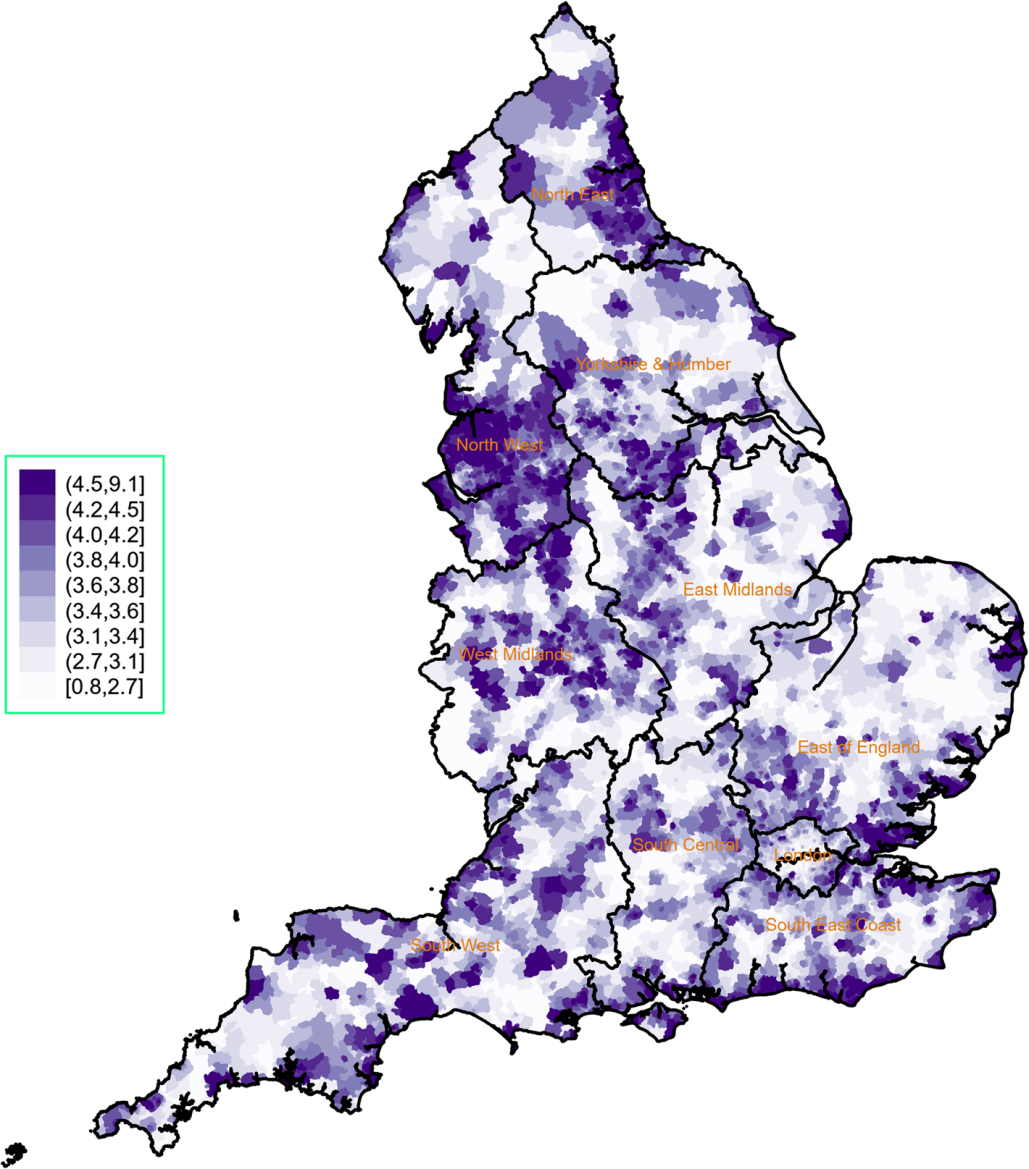
Ratio of chronic morbidity index over average primary medical care spending per patient (×1000), 2015-16.

The spatial variability of the CMI and average funding per person are presented in Figs. 2 and 3, respectively (regional spatial maps are also provided in Additional file 2: Appendix 2). We observed great variation in the CMI both across and within regions. Very high levels of morbidity were concentrated in large areas in the North East, the East Midlands and the East of England. London and parts of its neighbouring regions (South Central, South East and East of England) that are close to the capital consistently had the lowest CMI levels. For average funding per person, we also observed great regional variability, which did not necessarily match the CMI patterns. For example, there were very low levels of funding for many parts of the North West – and funding for the region as a whole was relatively low compared to other regions – whereas the overall CMI level in the region was the second highest nationally. We also calculated the ratio of CMI over average funding per person (×1000), which is plotted in Fig. 4 and highlights clusters of variability in every region and a pattern of higher ratios in the North West and the North East.

**Fig. 5:**
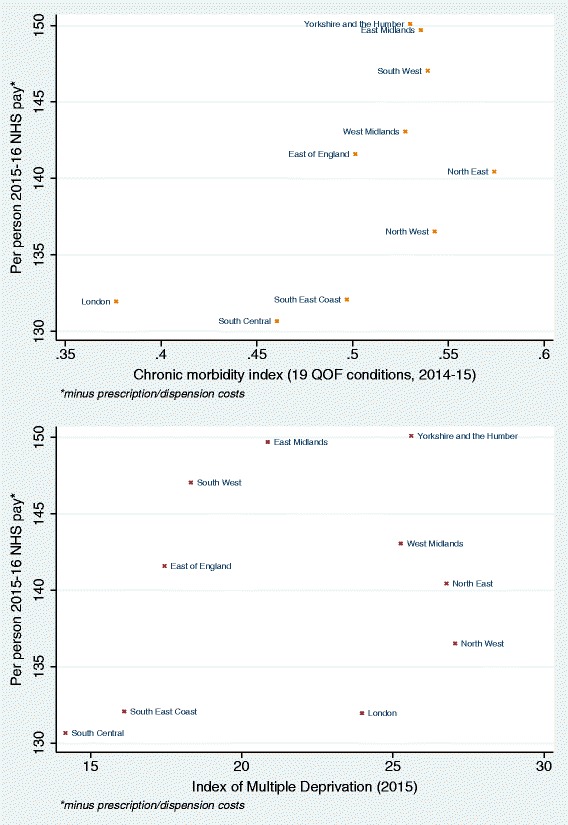
Scatter plot of average primary medical care spending for 2015-16 by chronic morbidity index (top) and the 2015 Index of Multiple Deprivation (bottom), across English regions, weighted for LSOA population sizes. LSOA Lower Layer Super Output Area, QOF Quality and Outcomes Framework

The associations between funding per person and CMI, and between funding per person and IMD, are illustrated at the regional level in Fig. 5 (plots at different geographic levels are provided in Additional file 3: Appendix 3). Increasing funding per person is associated with increasing levels of the CMI, with outliers at high and low levels of CMI (London, with the lowest level of CMI, has relatively high funding per person, and the North East and the North West regions, with the highest CMI levels, have relatively low funding). The association between funding per person and IMD is unclear, and IMD levels do not appear to be related to primary medical care funding at the regional level.

**Fig. 6:**
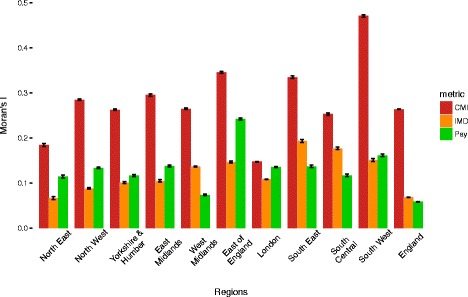
Spatial clustering (Moran’s *I* with 95% confidence intervals) for 2015-16 average primary medical care pay (minus prescription and dispensing costs, per person), the 2014/15 chronic morbidity index (CMI) and the 2015 Index of Multiple Deprivation (IMD), within each region and for the whole of England. CMI chronic morbidity index, IMD Index of Multiple Deprivation

The regional variability of spatial autocorrelation for the CMI, the IMD and funding is presented in Fig. 6. For the CMI, we observed high regional variability in spatial autocorrelation, ranging between 0.147 for London and 0.471 for the South West. This indicates that London has the least clustering of areas with similar levels of CMI, and the South West has the greatest clustering. For the IMD, spatial autocorrelation levels were lower and had less variation, ranging from 0.109 in London to 0.194 in the South East. Levels of spatial autocorrelation were also modest for funding, but there was greater variability between regions, ranging from 0.074 in the West Midlands to 0.242 in the East of England.

After adjusting for demographics, urbanity, the CMI and the IMD, there was large variability in funding between regions (Table 2). Compared to Yorkshire and the Humber, the region with the highest average funding per person, adjusted funding in the South East was £14.50 [95% confidence interval (CI), 13.38 to 15.62] lower per person. The CMI was a strong predictor of funding, with a 0.1-unit increase, i.e. from 0.513 (50th centile) to 0.613 (89th centile), associated with a £8.12 (95% CI, 7.71 to 8.54) increase in funding per person. The IMD was also a strong predictor with a 10-unit increase in IMD, i.e. from 17.4 (50th centile) to 27.4 (71st centile), associated with a £2.26 (95% CI, 2.06 to 2.45) increase in funding per person. The strongest independent predictor was urbanity, with rural areas associated with £35.71 (95% CI, 35.02 to 36.41) higher funding per person. Other large associations were observed for LSOAs with a large population over 60 and more females. An increase of 10% in patients over 60 within an LSOA (i.e. from 23.1% or the 50th centile to 33.1% or the 84th centile) was associated with a £2.54 (95% CI, 2.12 to 2.95) increase per person. The association for females is weaker than it appears because sex distribution is generally well balanced. An increase of 1% in females, i.e. an increase from 51% (50th centile) to 52% (73rd centile), was associated with a £0.49 (95% CI, 0.39, 0.59) decrease per person.

**Table 2:** Results from model A, linear regression at the LSOA level*†

	Coefficient	95% confidence interval		*p* value
Region				
Yorkshire and Humber	reference			
North East	−13.62	−14.93	−12.30	<0.001
North West	−12.47	−13.46	−11.47	<0.001
East Midlands	−3.42	−4.53	−2.32	<0.001
West Midlands	−6.59	−7.64	−5.54	<0.001
East England	−8.83	−9.88	−7.78	<0.001
London	−0.43	−1.64	0.78	0.484
South East	−14.50	−15.62	−13.38	<0.001
South Central	−12.38	−13.54	−11.21	<0.001
South West	−7.71	−8.78	−6.65	<0.001
Demographics				
Percentage aged 30–59, 2015	0.040	−0.013	0.093	0.140
Percentage aged 60 or over, 2015	0.254	0.212	0.295	<0.001
Percentage female, 2015	−0.489	−0.592	−0.387	<0.001
Percentage white British, 2011	−0.067	−0.084	-0.049	<0.001
Urbanity				
Rural LSOA	35.71	35.02	36.41	<0.001
Morbidity and deprivation				
Chronic morbidity index	81.22	77.08	85.36	<0.001
IMD 2015	0.226	0.206	0.245	<0.001
constant	118.34	112.80	123.89	<0.001

* 32,844 LSOAs (observations) with analytic weighting

† Adjusted *R*-squared = 38.85%

## Discussion

Primary-care funding in England is only modestly associated with morbidity burden, as measured by the CMI at the regional level, with the North East and North West having the highest levels of morbidity but only average levels of funding. We observed great variability in the CMI both within and between regions, with clusters of very high morbidity burden in the North East, East Midlands and East England. Spatial clustering of the CMI was high, especially for the South West, and was not associated with area deprivation in the patient locality, while funding was more uniformly distributed across space. Although morbidity, deprivation and age were strong predictors of funding, the strongest predictor was rurality, with less funding per person in urban centres. The model explained a reasonable level of variability in funding (39%), but even after adjusting for covariates, there was large variability between regions, which was also observed in relation to the strength of the associations between morbidity and funding, and between deprivation and funding.

### Strengths and limitations of the study

The strength of the study lies in the quality of the databases and their sizes. We investigated the whole of England and over 55 million people being served by a universal health system. Through their electronic health records, we calculated a measure of morbidity, the CMI, and linked it to funding per person. The CMI is designed to capture overall health needs for 19 common chronic conditions rather than multi-morbidity, but we would expect it to be a strong proxy for multi-morbidity.

The study has the potential for ecological fallacy, with practice-level information assigned to geographies. Although, we have assessed that risk and found it to be low in terms of deprivation (see Additional file 1: Appendix 1), the assignment could be improved by using age–sex stratification weights at the LSOA level. A second limitation is that ethnicity and urbanity information was available for 2011 and we necessarily assumed there has been little change over time. Although this will not be the case for all regions and LSOAs, the 2001 and 2011 versions of these variables were very strongly correlated, indicating little change over time. A third limitation is that we could not directly assess multi-morbidity in any form, due to the way the data are collected in England. It has been shown in Scotland that multi-morbidity is higher in deprived areas and is associated with higher demand but not additional funding [25]. Fourth, our analysis is dependent on accurate diagnosis and recording of conditions within primary care. There is a danger that any future system of resource allocation based on practice registers would incentivise practices to inflate these registers. Finally, to weight the regression models for LSOA sizes, we necessarily used standard regression models rather than spatial-autoregressive models, which may have affected our estimates. However, the estimated spatial autocorrelation for the whole of England is low and should not affect the precision of our model estimates.

The low-level geographical approach includes advantages as well as risks. It allows us to control analyses for population characteristics that are linked to the small area geography, especially area deprivation. It also enables us to plot detailed spatial maps to visualise the parameters of interest and to identify geographical clusters of high and low need. These analyses can inform future service reorganisations and, by identifying areas where the observed morbidity load is lower than expected, uncover possible under-diagnosis and unmet need [26].

### Findings

We failed to identify a strong association between the overall morbidity burden, as measured by the recorded prevalence of QOF conditions, and area deprivation. Although multi-morbidity levels are positively associated with deprivation [27], and the onset of multi-morbidity occurs much earlier in people living in the most deprived areas [28], our findings are not contradictory. First, mean age is lower in deprived inner-city areas, which offsets the unadjusted association between deprivation and morbidity. Second, although associations between deprivation and prevalence rates have been observed for conditions such as diabetes even when not adjusted for age [29], these balance out across the whole spectrum of conditions (at the practice level, Pearson’s rho = 0.05). However, this may also be an indication of under-diagnosis in more deprived areas and better case finding and recording in more affluent areas.

Regional variability in the CMI highlights the varying levels of prevalence for QOF conditions across England. The highest CMI levels were observed in the North East (median 0.59 conditions per person) and the lowest in London (median 0.38 conditions, reflecting its younger population). Our findings are broadly in agreement with previous reports for individual conditions at a higher geography [30], considering we report an aggregate measure. However, our low-geography mapping approach facilitates within-region investigations and the identification of geographical clusters of high disease prevalence. Large clusters of very high prevalence were observed for the North East, East Midlands and East England, and additional resources or reorganisation of services may be needed to serve these populations better [for example, extended opening hours or redistribution of general practitioners (GPs)]. Levels of spatial clustering were particularly high for the South West (there were no large high-prevalence clusters but there was a high level of spatial variation across the whole region), which could also inform the organisation of care. Importantly, although regional CMI levels were broadly associated with average primary medical care funding per person, the North East, North West and London appear to be outliers, with fewer than expected resources allocated to the North East and the North West, and more to London. In particular for Greater Manchester, and its very recently devolved health and social care spending, a £2bn a year funding gap is expected by 2020 if demand trends are not curbed and existing barriers to efficiency and effectiveness are not removed http://www.bmj.com/content/352/bmj.i1495.long. This imbalance between health need and resource allocation may be a contributing factor to the North–South divide in young adult mortality rates [31].

At the low-geography level, patient age, sex, ethnicity, morbidity (CMI), deprivation (IMD) and rurality explained a higher level of variation in funding than a previous model at the practice level for England [32]. More importantly, unlike in previous work at the practice level, we found the expected positive associations between funding and patient morbidity, deprivation and age. This may be an indication of the strength of our approach at the low geographical level, where area deprivation can be modelled in greater detail. However, regional variations in funding existed even after adjusting for all these parameters, with the South East, North East, North West and South Central having the lowest levels of adjusted funding. As expected, morbidity, deprivation and age were strong predictors of funding. Rurality was also a very strong independent predictor, with rural areas receiving an adjusted average of £35.70 more per patient. Prescribing and dispensing costs have been suggested as explaining this disparity, but these costs were excluded from our analyses. The higher cost seems to be at least partially driven by the smaller list sizes in practices located in rural areas (on average 831 fewer patients), while staffing levels are similar in both rural (average full-time equivalent of 4.2 GPs and 2.3 nurses) and urban settings (average full-time equivalent of 4.1 GPs and 2.1 nurses).

## Conclusions

To meet societal goals of providing equitable health care, funding for primary-care systems must be distributed according to need, fully accounting for the impact of deprivation. We have described the morbidity burden in England at a low geographical level using routinely collected administrative data. Not only have we identified unexplained regional variation in common morbidities, we have also found evidence that the current allocation of resources to primary care does not account for all important health needs. More optimal resource utilisation in primary care in the UK is required [25], and better use of the wealth of information resources already available could help to achieve this aim through the design of fairer resource allocation formulae. It is, therefore, vital that the disease registers introduced as part of the QOF are retained and updated when the scheme is phased out. Similar information is also routinely collected in other developed countries, and the methods we have described can be highly relevant, both in identifying disease clusters or under-diagnosis, and in matching resources to need. Findings from such analyses are used in countries that use public-sector allocation mechanisms, whether central (e.g. Ireland and France) or devolved (e.g. Denmark, Sweden and Spain), to directly inform resource allocation. In countries with insurance-based mechanisms (e.g. France, Germany and the Netherlands), analyses can be used to identify mismatches between allocation and need, which may require government intervention through risk equalisation schemes [33] or alternative mechanisms. This will require difficult decisions about the fundamental aims of resource allocation and – within a limited health budget – a commitment to tackling oversupply as well as undersupply [34].

## Additional files

Additional file 1: Appendix 1. Details of methods and additional analyses. (DOCX 47 kb)

Additional file 2: Appendix 2. Spatial maps by English region. (DOCX 3327 kb)

Additional file 3: Appendix 3. Scatter plots for deprivation vs. funding and morbidity vs. funding. (DOCX 325 kb)
