# Supplementary material for: Chronic morbidity, deprivation and primary medical care spending in England in 2015-16: a cross-sectional spatial analysis
Source: BMC Med. 2018 Feb 14;16:19. doi: 10.1186/s12916-017-0996-0 (PMC5812046; doi:10.1186/s12916-017-0996-0)
Supplement: Supplementary file 1 — Details of methods and additional analyses. (DOCX 47 kb) [file 12916_2017_996_MOESM1_ESM.docx]

# Online appendix 1: details on methods and additional analyses

# Data collection

## English Indices of Deprivation

In England, the most complete and widely used approach to quantify deprivation and affluence is through the English Indices of Deprivation and their aggregate score the Index of Multiple Deprivation (IMD), with the latest available update released in 2015.^1^ The measure quantifies relative deprivation across seven domains: income, employment, education and skills, health and disability, crime, barriers to housing and services, and living environment. Deprivation scores are calculated and assigned to very low UK geographical units. The overall IMD is calculated as a weighted mean across the seven domains, with income and employment deprivation given the largest weight (22.5% each), followed by health and education deprivation (13.5% each), and with the other three domains given equal weights (9.3%).

## Lower Super Output Areas

The low geographical units to which the indices of deprivation are assigned are called lower super output areas (LSOAs) and they are designed to contain around 1500 inhabitants, on average. Following the 2011 census, there were 32,844 English LSOAs.^2^ Ethnicity information was collected through the census.^3^ Urbanity information was also updated following the census,^4^ and we used a rural vs urban dichotomy for simplicity, with settlements with 10,000 people or more defined as urban. Census-adjusted population estimates over time, by age groups and sex and for each English LSOA, were obtained from the Office of National Statistics.^5^ Spatial coordinates for the 2011 LSOAs were obtained from the ONS open geography portal.^6^ We used digital vector boundaries generalised to 20 meters and clipped to the coastline to reduce size and improve visualisation. To allow for comparisons within England, we organised LSOAs into 10 regions: North East, North West, Yorkshire & the Humber, East Midlands, West Midlands, East of England, London, South East Coast, South Central and South West.^7^

## Chronic morbidity index (CMI)

The Quality and Outcomes Framework (QOF), since its introduction in 2004,^8^ has underpinned high quality of recording in primary care.^9^ Under the QOF umbrella, recording, management and treatment of large number of clinical domains was financially and reputationally incentivised. The number of domains varied over time with a maximum of 24 clinical domains being included in the scheme for the 2013-14 financial year.^10^ We focused on financial year 2014-15, as the most relevant in linking to the latest available primary care payment data (2015-16). The underlying hypothesis is that health need drives payments to primary care and morbidity burden on a particular year should be associated with payments to practice in the following year. In 2014-15, 21 clinical domains were incentivised under the QOF and we focused on 19 that reflected chronic conditions, thus excluding Cardiovascular Disease Primary Prevention and Obesity. The 19 conditions were: Atrial Fibrillation, Asthma, Cancer, Coronary Heart Disease, Chronic Kidney Disease (for those aged 18 or over), Chronic Obstructive Pulmonary Disease, Dementia, Depression (18 or over), Diabetes both types (17 or over), Epilepsy (18 or over), Heart Failure, Hypertension, Learning Disability (18 or over), Severe Mental Illness, Osteoporosis (50 or over), Peripheral Artery Disease, Palliative Care, Rheumatoid Arthritis (16 or over) and Stroke.

General practices use specific annually issued coding guidelines to record the domain registers, i.e. the number of patients included in each domain. The current coding system uses a clinical vocabulary based on Read codes, ^9^ but a switch to SNOMED CT is underway. The domain registers for 2014-15 were extracted on the last day of the financial year (31 March 2015). For each of the 7,779 practices contributing to the QOF we calculated the total sum of all condition registers (since multimorbidity at the patient level cannot be accounted in existing national administrative systems) and also the chronic morbidity index (CMI), the total register over the practice list size. Next, using the total register and a database that attributes general practice patients to LSOAs using their residential postcode (attribution dataset),^11^ we estimated the CMI at the LSOA level as a weighted mean. For example, if an LSOA was served by two practices, one looking after 900 patients with a CMI of 0.3 and the second 500 patients with 0.5, the LSOA CMI would be ((900*0.3)+(500*0.5))/1400=0.371. The key underlying assumption of the measure is that the distribution of practice morbidity to LSOAs is uniform (see limitations section). A very small number of practice registered patients (in Jan 2015: 20,377 of 56,924,424 patients, or 0.036%) not attributed to an LSOA because of a missing postcode in the record, due to homelessness or another reason, were excluded from the process.

Although access to health care is free in the UK, not all people in the UK are registered with primary care and not all general practices participate in the QOF. However, over 95% of the UK population is estimated to be registered with a practice, and over 99% of registered patients attend practices participating in the QOF.^12^ Therefore, we argue that the CMI is a measure that can successfully capture the health of the population of England at a low geographical level.

## Payments to general practice

We obtained data on National Health Service payments to general practice for the financial year 2015-16 and for the whole of England.^13^ The database included details on all payments made to each general practice under various different remuneration schemes (including Global Sum, the Minimum Practice Income Guarantee (MPIG), Balance of PMS expenditure, Quality Outcomes Framework (QOF) and Enhanced Services; but not including invoices raised by providers that were paid directly from other sources like Local Authority Public Health funding). We calculated the total payment made but also the total payment made minus prescribing and dispensing fee payments, since most practices are not dispensing medications and we aimed to obtain comparable payment estimates across practices. As for the CMI, we used the attribution dataset to transpose the financial data from general practice to the LSOA level,^11^ under the same assumptions. Per person LSOA payment estimates were calculated as a weighted mean of the average payment within each contributing practice, using two approaches. The first approach used the attribution population (i.e. from practice list sizes) as the denominator. The second approach used the 2015 LSOA population estimates as the denominator, aiming to adjust for areas with under-registration and, especially, over-registration and thus provide more realistic costs (generally higher than reported elsewhere).^14^

# Additional Analyses

At the LSOA level (with 32,844 data points), we used two linear regression models to quantify the strength of association between the outcome of interest, average primary care pay per person and: region, demographic characteristics (age, sex and ethnicity), urbanity, the CMI and the IMD. The first model (A) did not include interaction terms (presented in the main paper), while the second model (B) included interaction terms between region and IMD, and also region and CMI. The role of the interaction terms was to evaluate whether the association of each of the two key covariates with primary care pay varied across regions. Using model B estimates and the *margins* command in Stata, we estimated and plotted average levels of pay at various levels of IMD and CMI, within each region. Both models were weighted for the size of each LSOA, using 2014 population estimates.

# Additional Results

Results from regression model B show that the adjusted associations between the CMI and pay, and the IMD and pay vary across regions (Table A1). This regional variability can be observed in Figure A1. There we see that higher levels on the CMI mean higher pay in all regions, except London. However, the strength of the (largely) positive association varies, with the steeper slope observed for East England and the least steep for the North West. Positive associations were also observed between the IMD and pay in all regions, except the East Midlands. The slopes in this graph are much gentler, reflecting the weaker associations (compared to the CMI and pay). Nevertheless, we still observe regional variability with the steeper slopes observed for London, the North West and the North East.

# Limitations: ecological fallacy assessment

The key underlying assumption in our analyses is that the distribution of practice data to attributed LSOAs is uniform, when it may be the case that more deprived LSOAs carry higher rates of morbidity and higher payment, which would lead to a form of ecological fallacy. An average practice (7304 patients in 2014-15) serves under 5 LSOAs (average of 1654 people in 2014) and therefore practices located in deprived areas tend to serve populations residing in deprived areas. However, some variability is present in IMD patient residence within each practice. Across all practices, the mean standard deviation of weighted patient location IMD was 10.2, which would take the IMD from 14.4 (50^th^ centile) to 24.6 (66^th^ centile) across all LSOAs. Thus, under the assumption that morbidity and payments are not uniformly distributed across LSOAs, and deprivation is a confounding factor, our regression models would underestimate the associations between morbidity, deprivation and payments (regional comparisons would be unaffected). On the other hand, variability between practices in weighted patient residence IMD also exists, and the standard deviation of the mean practice IMD was 11.7. This between-practice IMD variability (which implies that practices in more deprived locations serve patients living in more deprived areas) was not masked in the models and should be enough to identify an existing association between deprivation and the CMI. To scrutinize the confounding potential of deprivation in our uniform attribution assumption even further, we investigated the associations between pay, deprivation and morbidity at the practice level. These were non-existent, except for the association between pay and morbidity, indicating that practices in poorer areas are not paid more and do not have higher morbidity levels, corroborating our uniform attribution assumption. We conclude, regarding this potential limitation, that ecological fallacy is unlikely.

# References

1. Communities and Local Government. The English Indices of Deprivation 2015: Technical Report. Government DfCaL, 2015. <https://www.gov.uk/government/publications/english-indices-of-deprivation-2015-technical-report>

2. Office for National Statistics. Changes to Output Areas and Super Output Areas in England and Wales, 2001 to 2011. ONS, 2012:13. <http://www.ons.gov.uk/ons/guide-method/geography/products/census/report--changes-to-output-areas-and-super-output-areas-in-england-and-wales--2001-to-2011.pdf>

3. Office of National Statistics. Census 2011 Table Links. <https://www.nomisweb.co.uk/census/2011/quick_statistics>

4. Bibby P, Brindley P. Urban and rural area definitions for policy purposes in England and Wales: Methodology. *Government Statistical Service, London* 2013.

5. Office for National Statistics. Super Output Area mid-year population estimates for England and Wales, Mid-2011 (Census Based). 2016. <http://www.ons.gov.uk/ons/publications/re-reference-tables.html?edition=tcm%3A77-285629>

6. Office for National Statistics. Open Geography Portal. 2016. <http://geoportal.statistics.gov.uk/>

7. Department of Health. Strategic Health Authority Configurations. <https://web.archive.org/web/20070205232558/http://www.dh.gov.uk/assetRoot/04/13/37/60/04133760.pdf>

8. Roland M. Linking physicians' pay to the quality of care--a major experiment in the United kingdom. *N Engl J Med* 2004;351:1448-54.

9. Olier I, Springate DA, Ashcroft DM, Doran T, Reeves D, Planner C, et al. Modelling Conditions and Health Care Processes in Electronic Health Records: An Application to Severe Mental Illness with the Clinical Practice Research Datalink. *Plos One* 2016;11.

10. Health & Social Care Information Centre. The Quality and Outcomes Framework. 2013. <http://www.ic.nhs.uk/qof>

11. NHS Digital. Numbers of Patients Registered at a GP Practice - Jan 2016. <http://content.digital.nhs.uk/article/2021/Website-Search?productid=20032&q=Numbers+of+Patients+Registered+at+a+GP+Practice&sort=Relevance&size=10&page=1&area=both#top>

12. Health & Social Care Information Centre. Quality and Outcomes Framework Achievement, prevalence and exceptions data, 2012/13: Frequently asked questions. HSCIC, 2013:20. <http://www.hscic.gov.uk/catalogue/PUB12262/qual-outc-fram-freq-aske-ques-2012-13-gui.pdf>

13. NHS Digital. NHS Payments to General Practice, England, 2015/16. <http://content.digital.nhs.uk/catalogue/PUB21318>

14. Levene LS, Baker R, Wilson A, Walker N, Boomla K, Bankart MJG. Population health needs as predictors of variations in NHS practice payments: a cross-sectional study of English general practices in 2013-2014 and 2014-2015. *Brit J Gen Pract* 2017;67:E10-E19.

Table A1: Results from model B, linear regression at the LSOA level, with region-morbidity and region-IMD interactions*†‡

|  | coefficient | 95% Confidence Interval | | p-value |
| --- | --- | --- | --- | --- |
| Region | | | | |
| Yorkshire & Humber | reference | | | |
| North East | -7.73 | -17.55 | 2.08 | 0.122 |
| North West | 1.99 | -4.78 | 8.75 | 0.565 |
| East Midlands | -23.55 | -30.51 | -16.59 | 0.000 |
| West Midlands | -2.96 | -10.35 | 4.42 | 0.432 |
| East England | -37.91 | -44.36 | -31.46 | 0.000 |
| London | 43.14 | 36.65 | 49.64 | 0.000 |
| South East | -20.58 | -27.40 | -13.77 | 0.000 |
| South Central | -1.75 | -8.69 | 5.20 | 0.622 |
| South West | -4.64 | -11.59 | 2.32 | 0.191 |
| Demographics† | | | | |
| % aged 30-59, 2015 | -0.01 | -0.06 | 0.05 | 0.835 |
| % aged 60 or over, 2015 | 0.21 | 0.17 | 0.25 | 0.000 |
| % Female, 2015 | -0.45 | -0.55 | -0.35 | 0.000 |
| % White British, 2011 | -0.04 | -0.06 | -0.03 | 0.000 |
| Urbanity† | | | | |
| Rural LSOA | 34.47 | 33.78 | 35.16 | 0.000 |
| Morbidity and deprivation† | | | | |
| Chronic morbidity index | 87.27 | 78.23 | 96.31 | 0.000 |
| IMD 2015 | 0.18 | 0.14 | 0.22 | 0.000 |
| constant | 115.37 | 108.55 | 122.18 | 0.000 |
| Region#Morbidity interaction‡ | | | | |
| Yorkshire & Humber | reference | | | |
| North East | -15.60 | -32.63 | 1.44 | 0.073 |
| North West | -33.64 | -45.72 | -21.57 | 0.000 |
| East Midlands | 44.21 | 31.80 | 56.61 | 0.000 |
| West Midlands | -9.12 | -22.28 | 4.04 | 0.174 |
| East England | 62.15 | 50.03 | 74.28 | 0.000 |
| London | -123.41 | -137.00 | -109.82 | 0.000 |
| South East | 14.53 | 1.59 | 27.47 | 0.028 |
| South Central | -24.68 | -38.22 | -11.15 | 0.000 |
| South West | -1.92 | -14.48 | 10.65 | 0.765 |
| Region#IMD interaction‡ | | | | |
| Yorkshire & Humber | reference | | | |
| North East | 0.10 | 0.03 | 0.18 | 0.006 |
| North West | 0.14 | 0.08 | 0.19 | 0.000 |
| East Midlands | -0.17 | -0.24 | -0.11 | 0.000 |
| West Midlands | 0.05 | -0.01 | 0.11 | 0.089 |
| East England | -0.12 | -0.19 | -0.05 | 0.001 |
| London | 0.18 | 0.12 | 0.24 | 0.000 |
| South East | -0.08 | -0.16 | 0.00 | 0.041 |
| South Central | 0.05 | -0.03 | 0.14 | 0.210 |
| South West | -0.12 | -0.20 | -0.05 | 0.001 |

* 32844 LSOAs (observations) with analytic weighting, adjusted R-squared=40.89%

† Coefficients reported are for the reference region only, Yorkshire & the Humber

‡ Differences to the coefficients reported for the reference region

Figure A1: Cost estimates (and 95% CIs) by region from model B, at various levels for the chronic morbidity index (top) and the index of multiple deprivation (bottom)*

|  |
| --- |
|  |

* Corresponding to 10^th^, 25^th^, 50^th^, 75^th^ and 90^th^ centile for the chronic morbidity index (0.375, 0.445, 0.513, 0.568 and 0.614) and deprivation as measured by the Index of Multiple Deprivation 2015 (5.7, 9.7, 17.4, 30.1 and 44.6)
