# Supplementary material for: Chronic morbidity, deprivation and primary medical care spending in England in 2015-16: a cross-sectional spatial analysis
Source: BMC Med. 2018 Feb 14;16:19. doi: 10.1186/s12916-017-0996-0 (PMC5812046; doi:10.1186/s12916-017-0996-0)

# **Online appendix 2: Spatial maps by English region**

Figure B1: North East


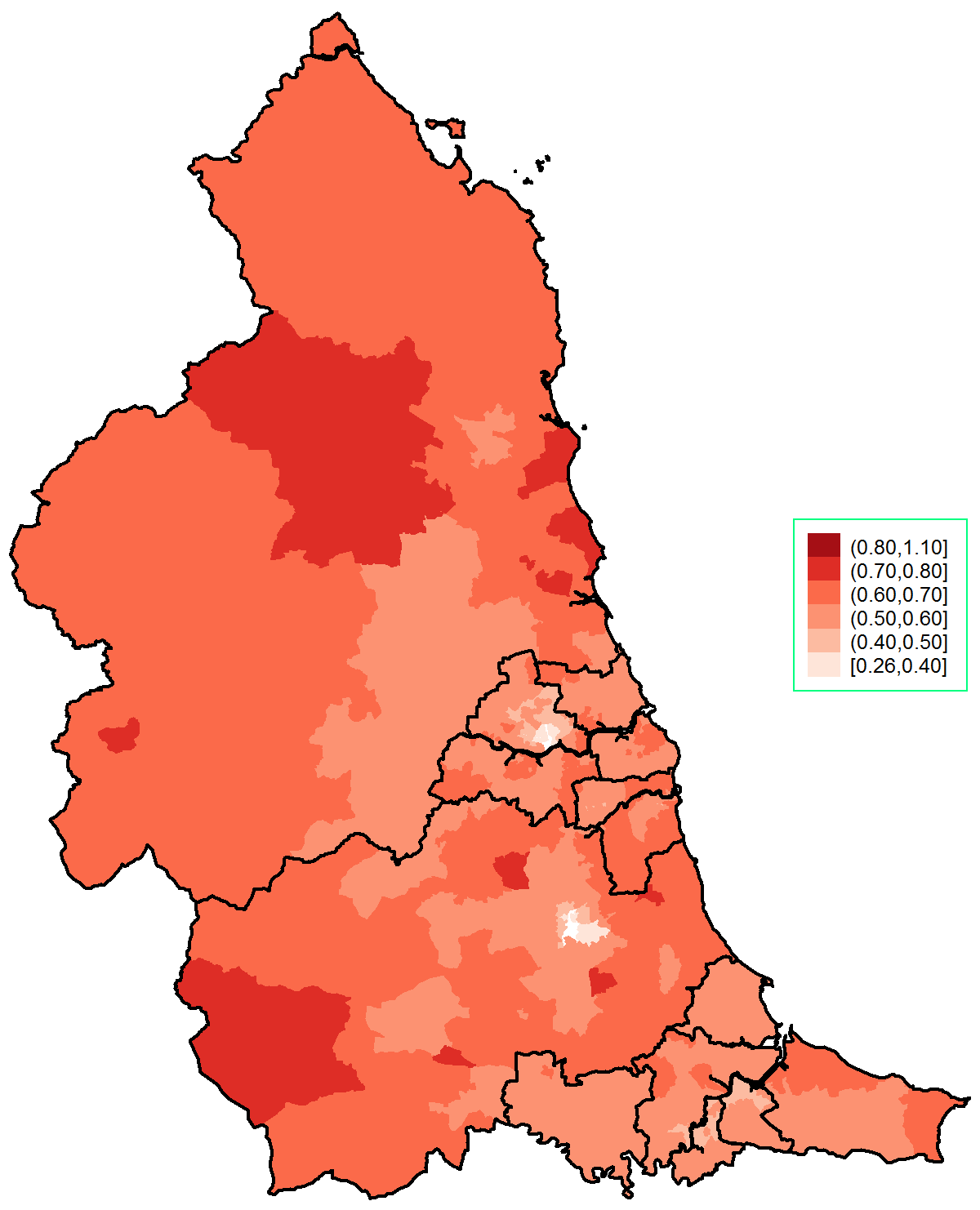


Figure B2: North West


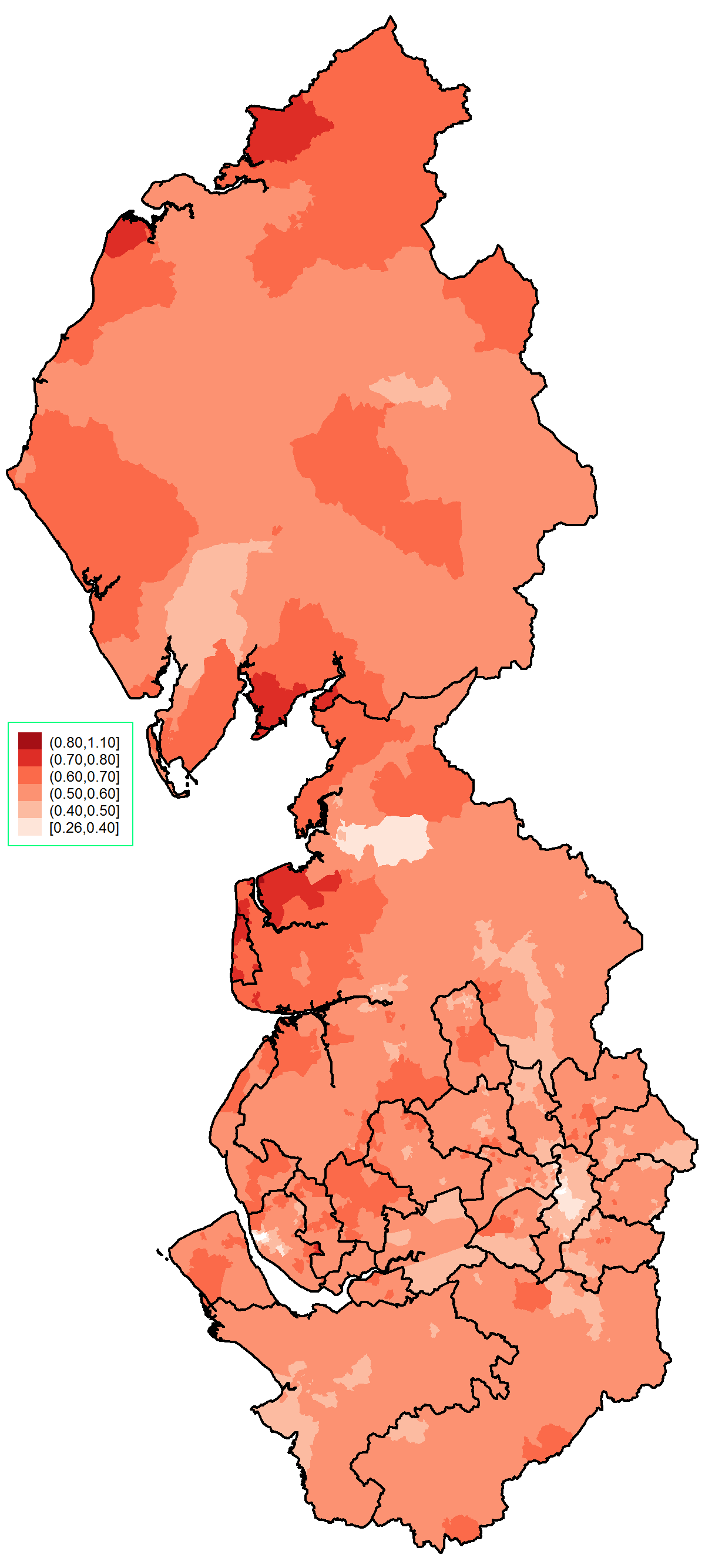


Figure B3: Yorkshire and the Humber


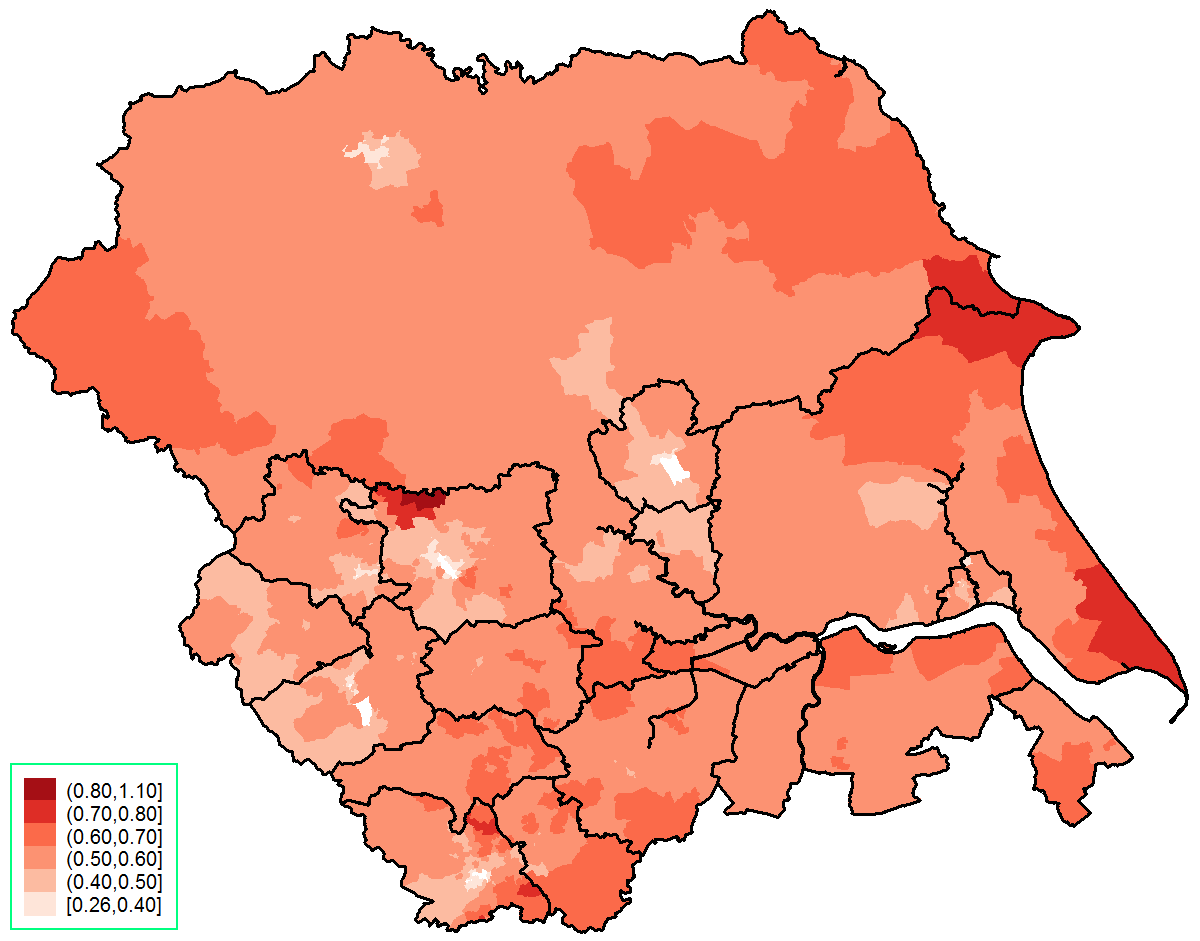


Figure B4: East Midlands


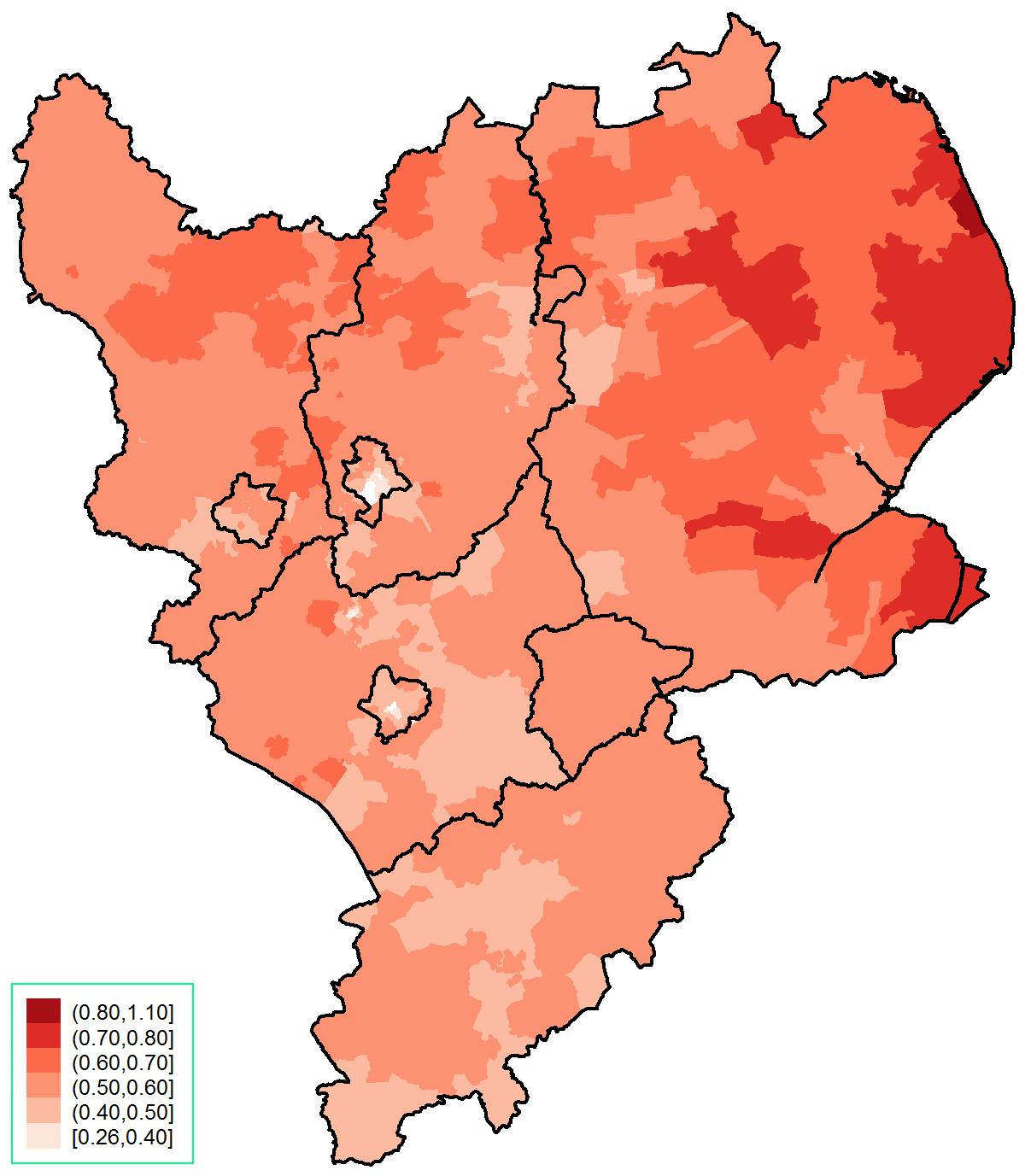


Figure B5: West Midlands


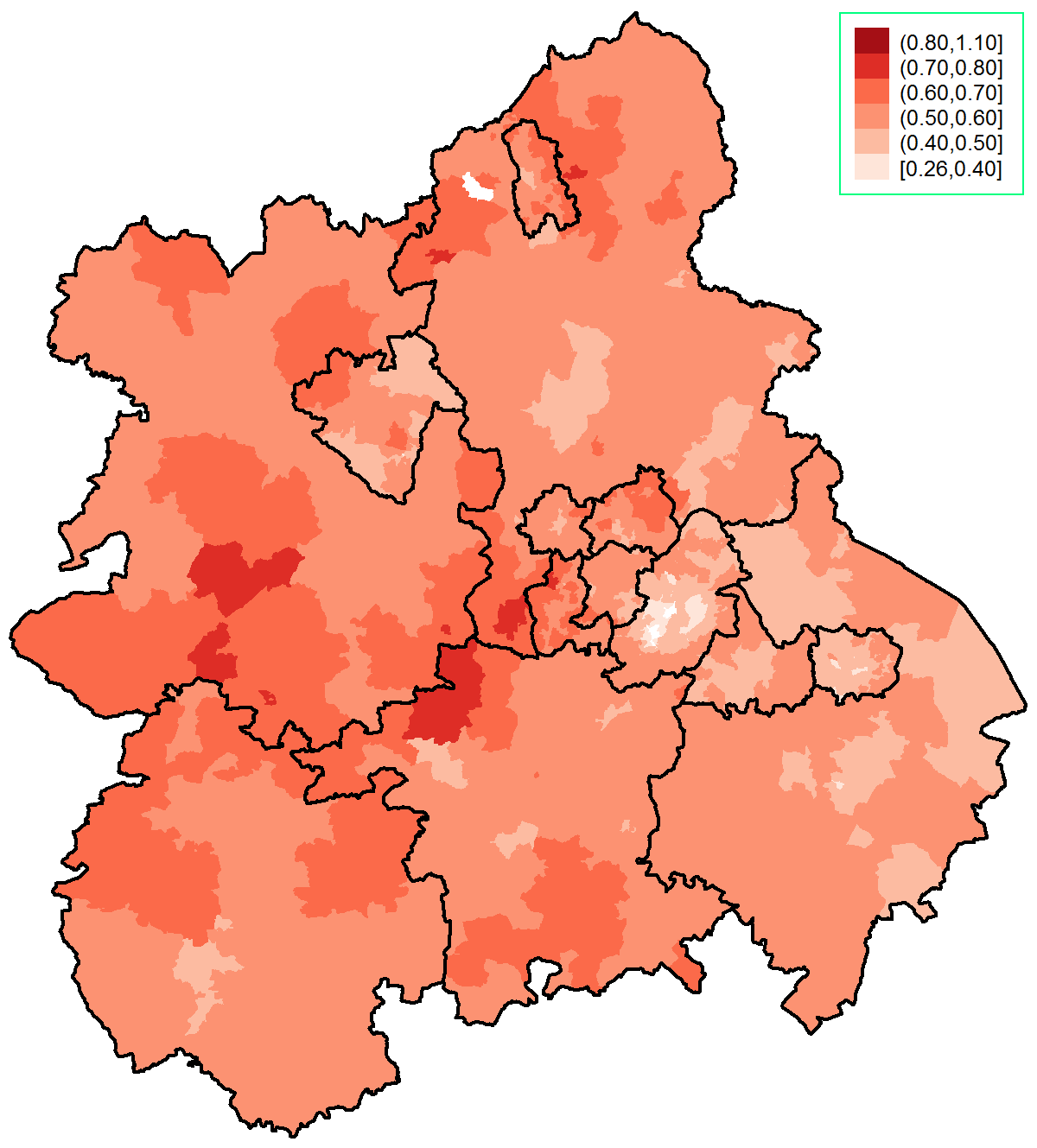


Figure B6: East of England


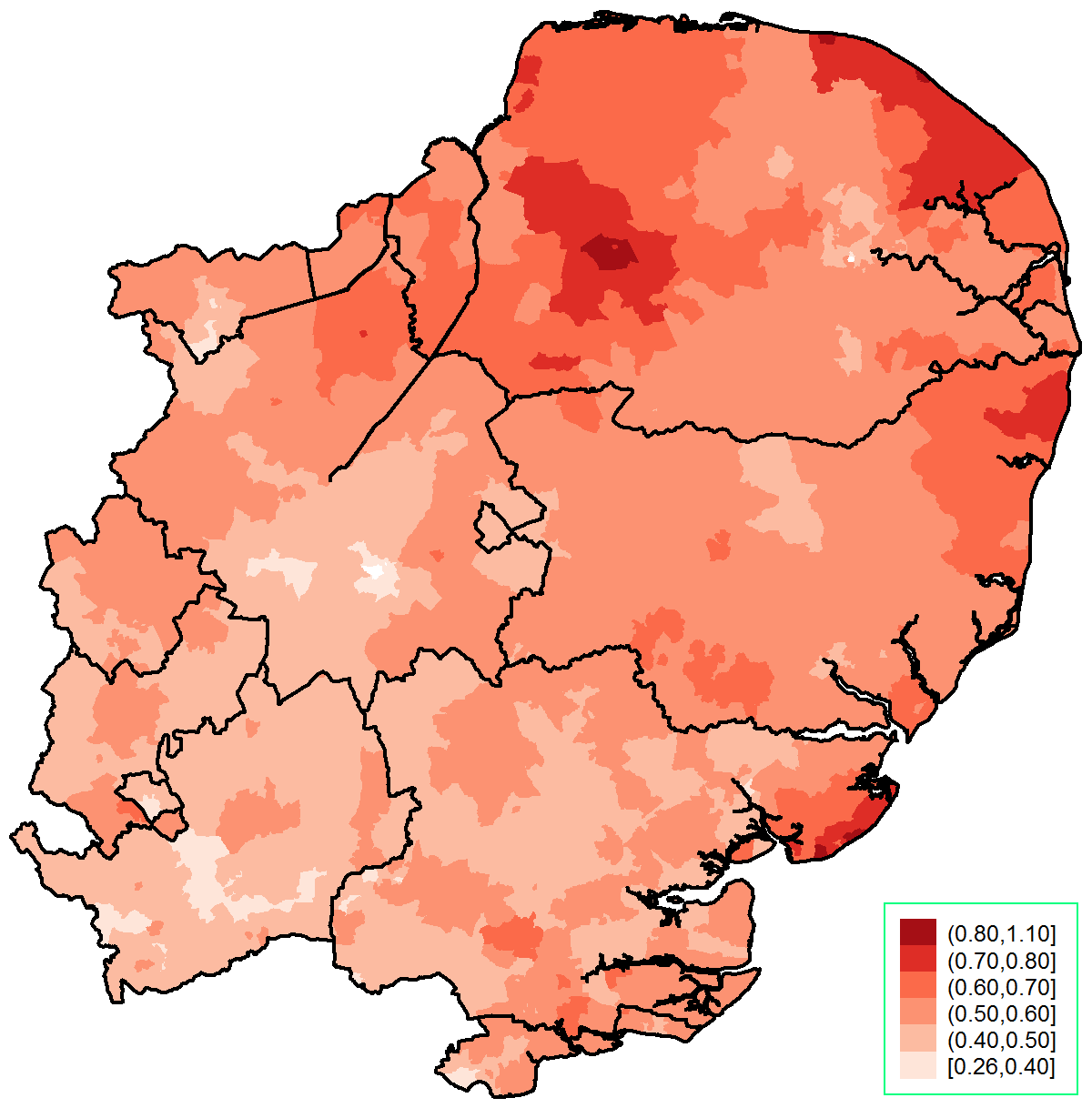


Figure B7: London


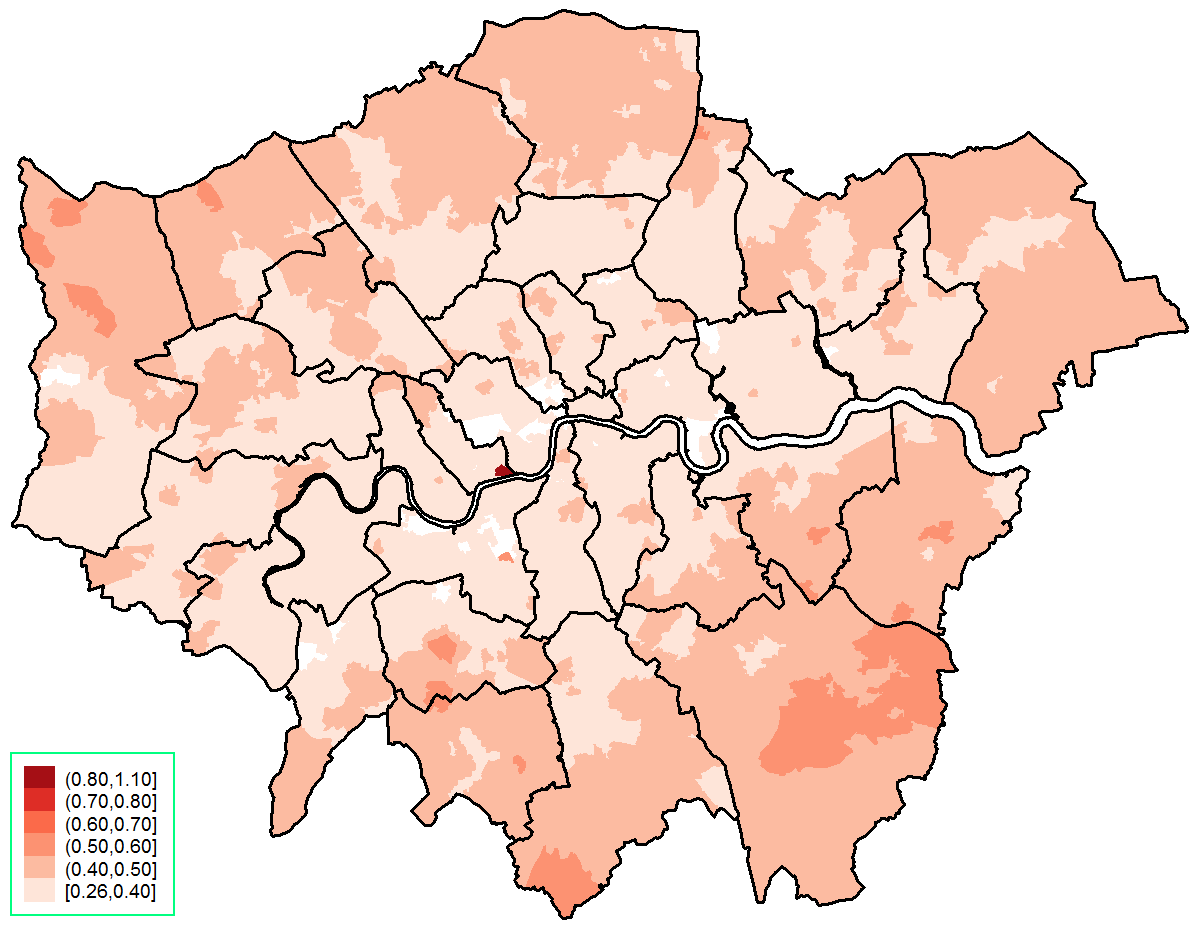


Figure B8: South East Coast


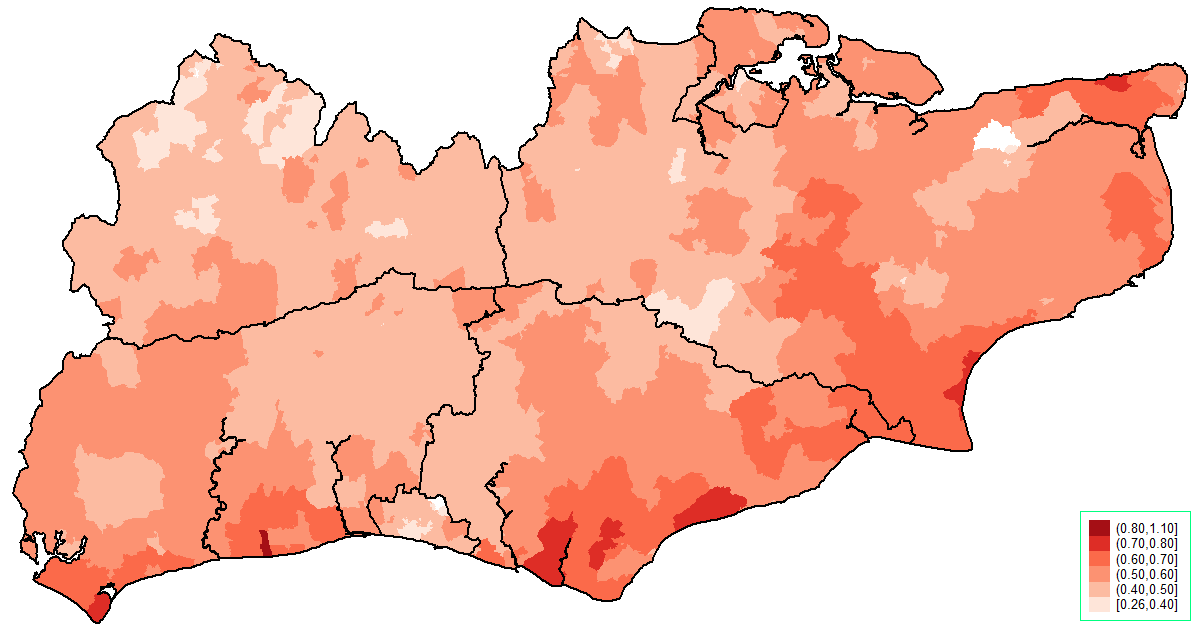


Figure B9: South Central


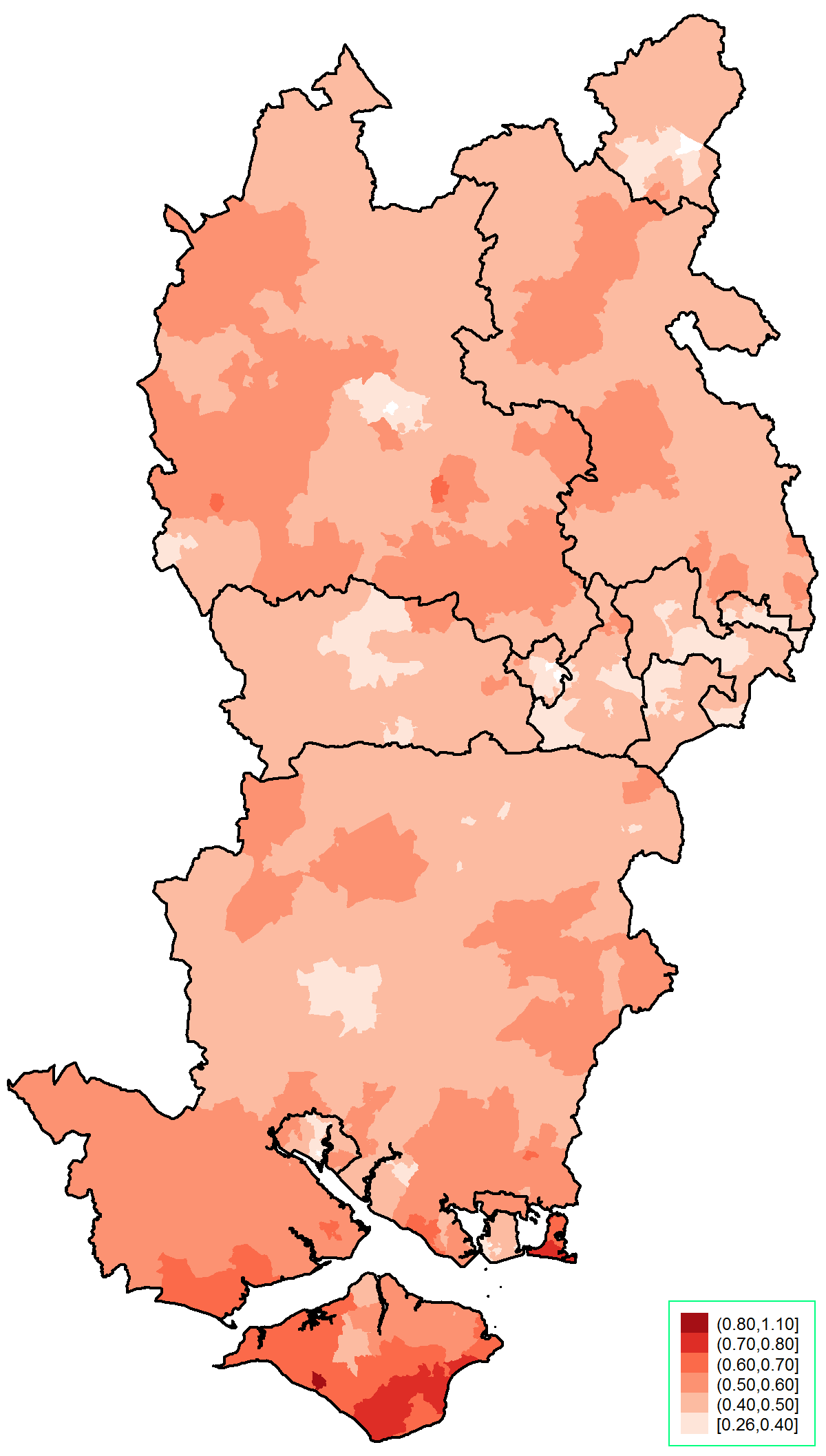


Figure B10: South West


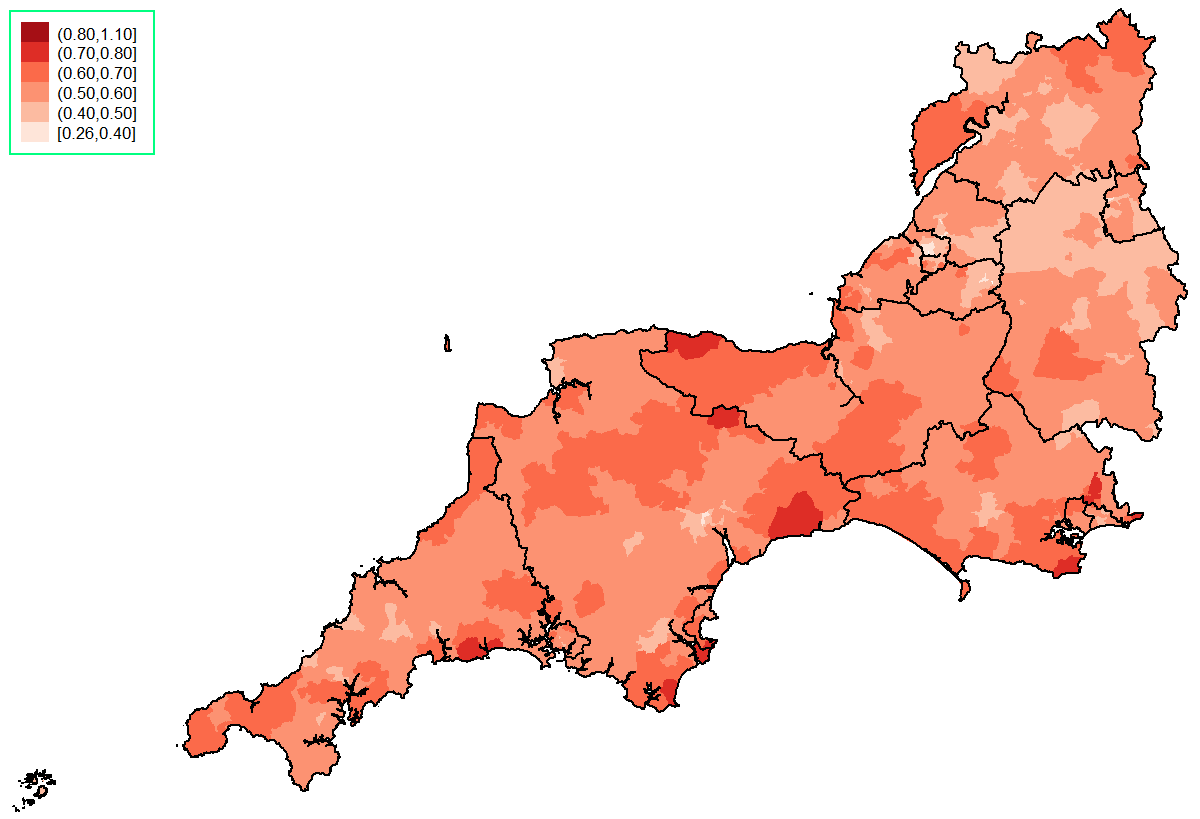


Figure B11: Greater Manchester (North West sub-region)


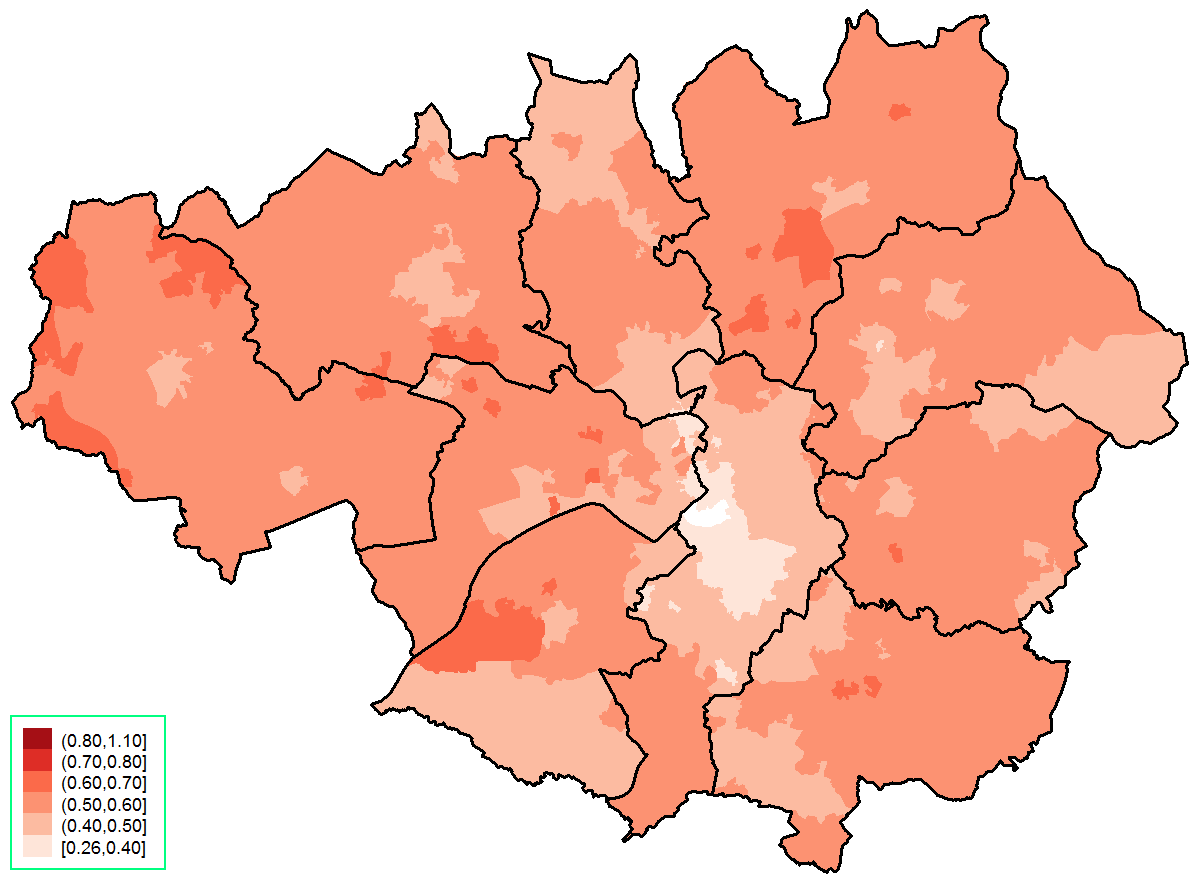


Figure B12: Birmingham (West Midlands sub-region)


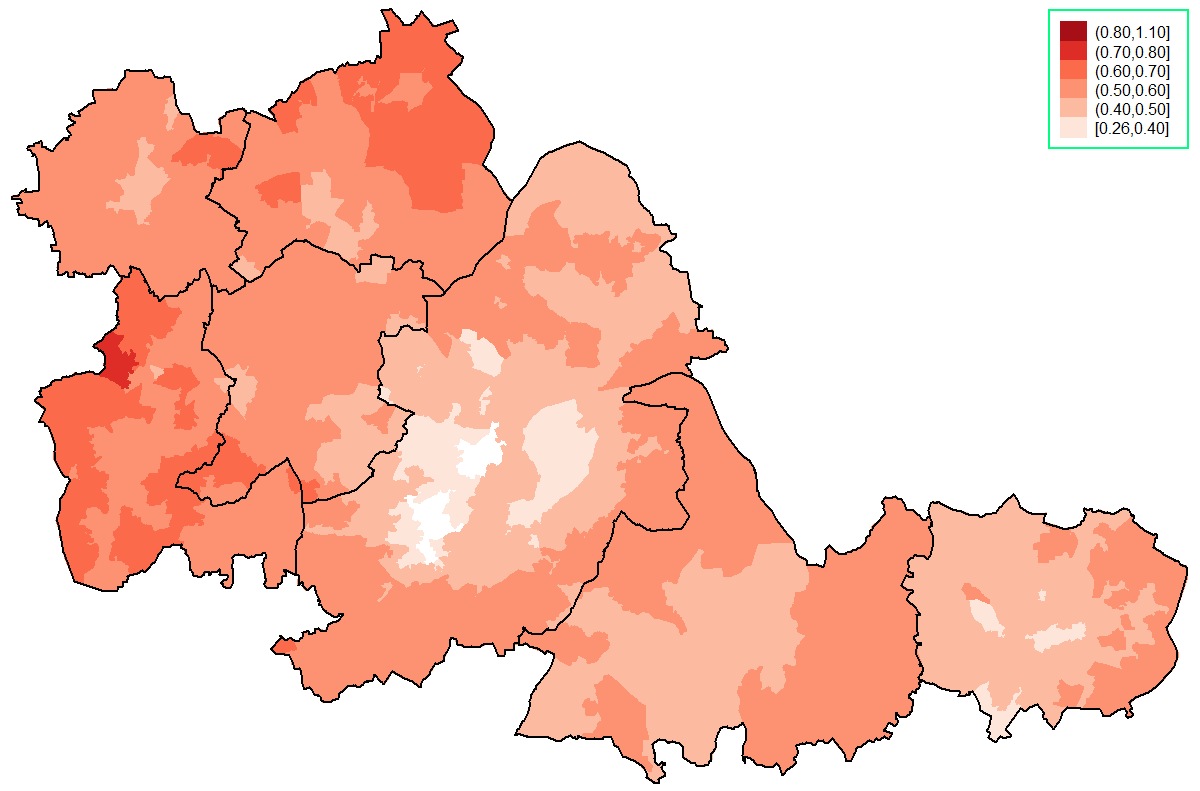


Figure B13: North East


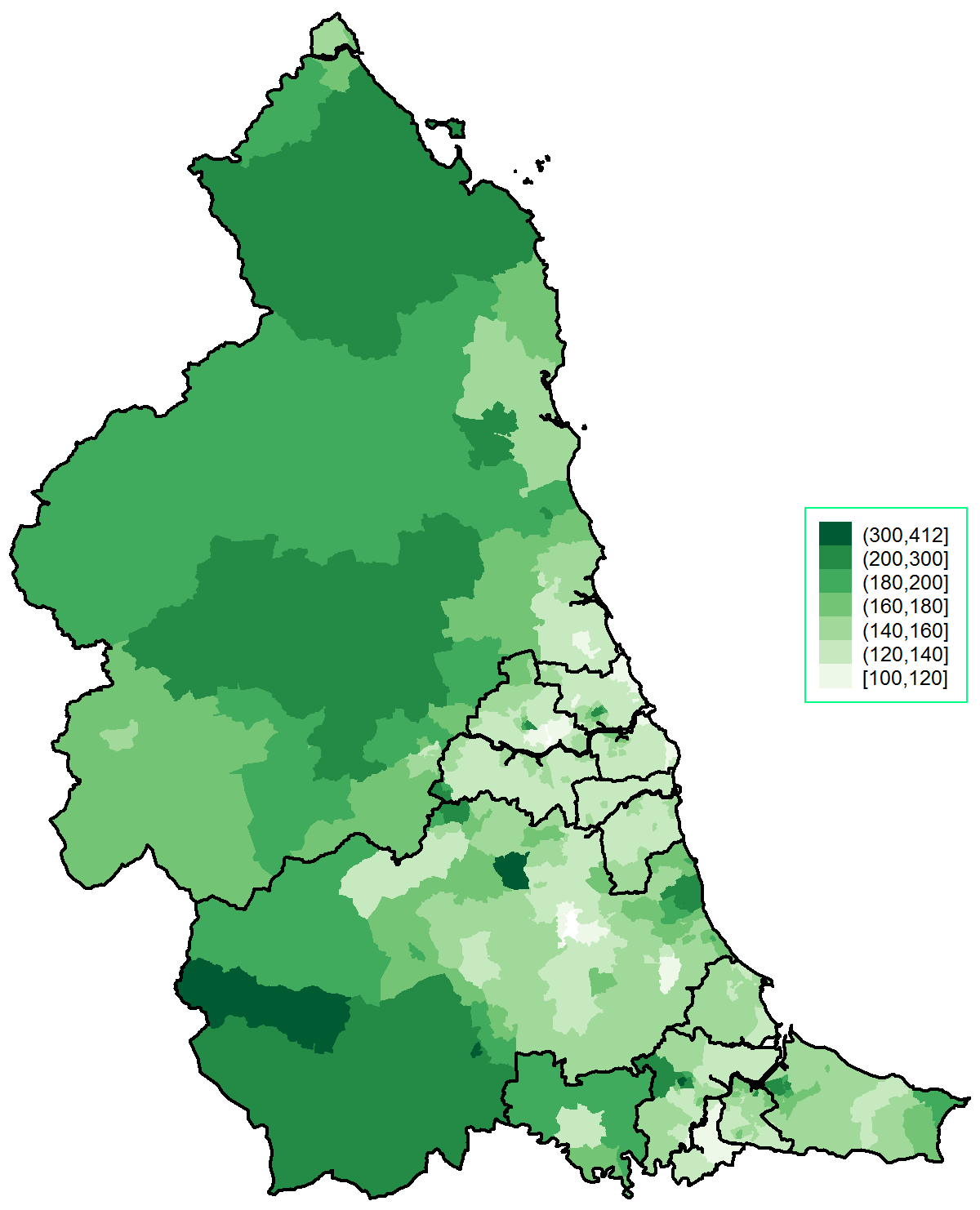


Figure B14: North West


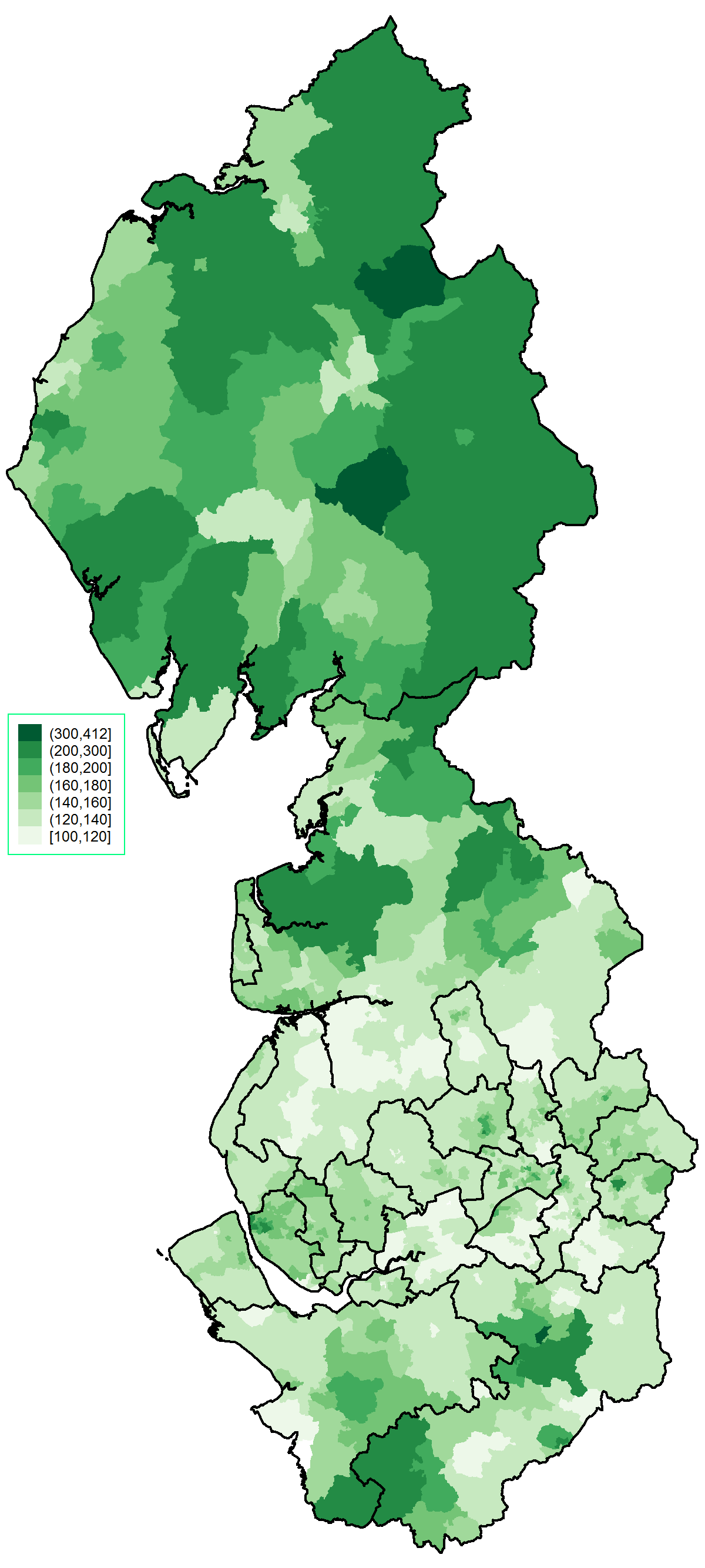


Figure B15: Yorkshire and the Humber


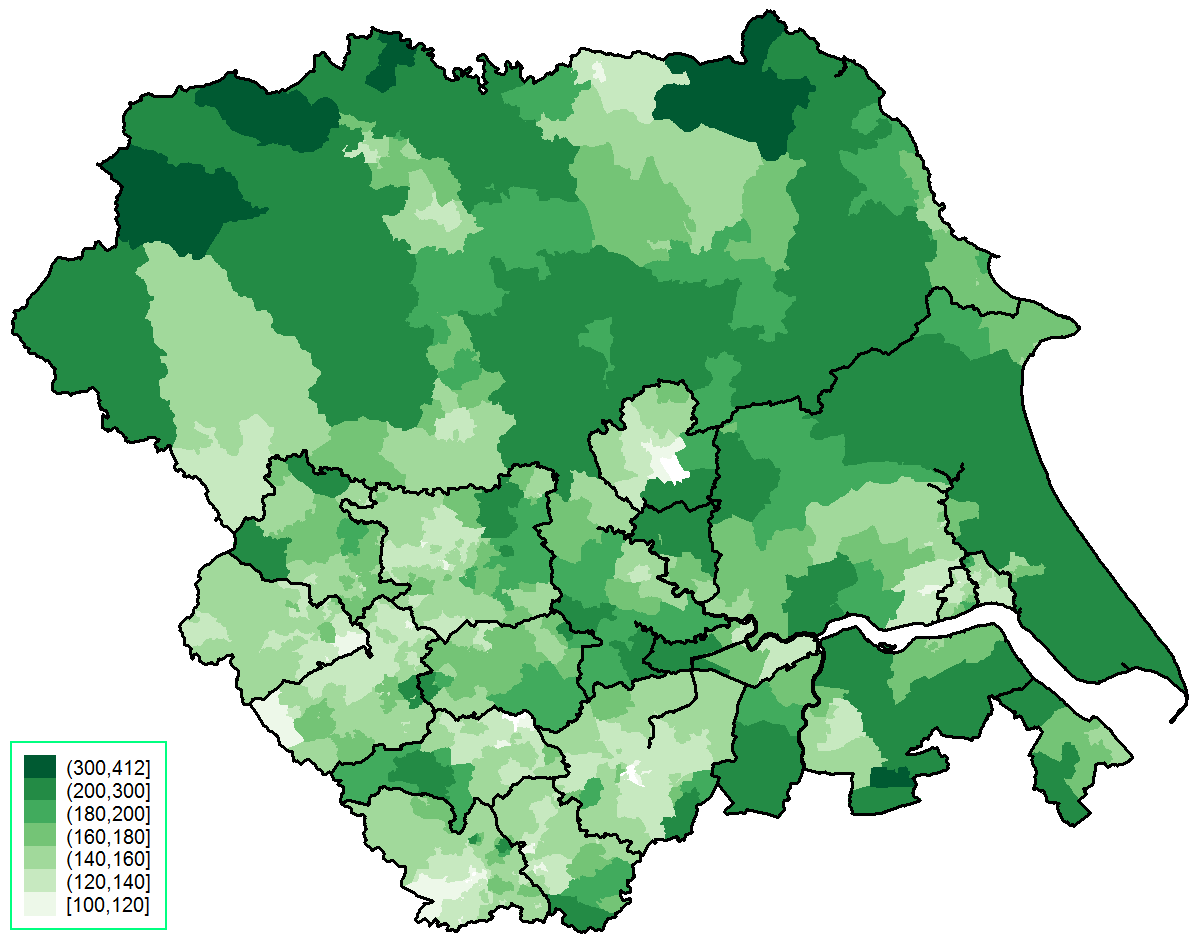


Figure B16: East Midlands


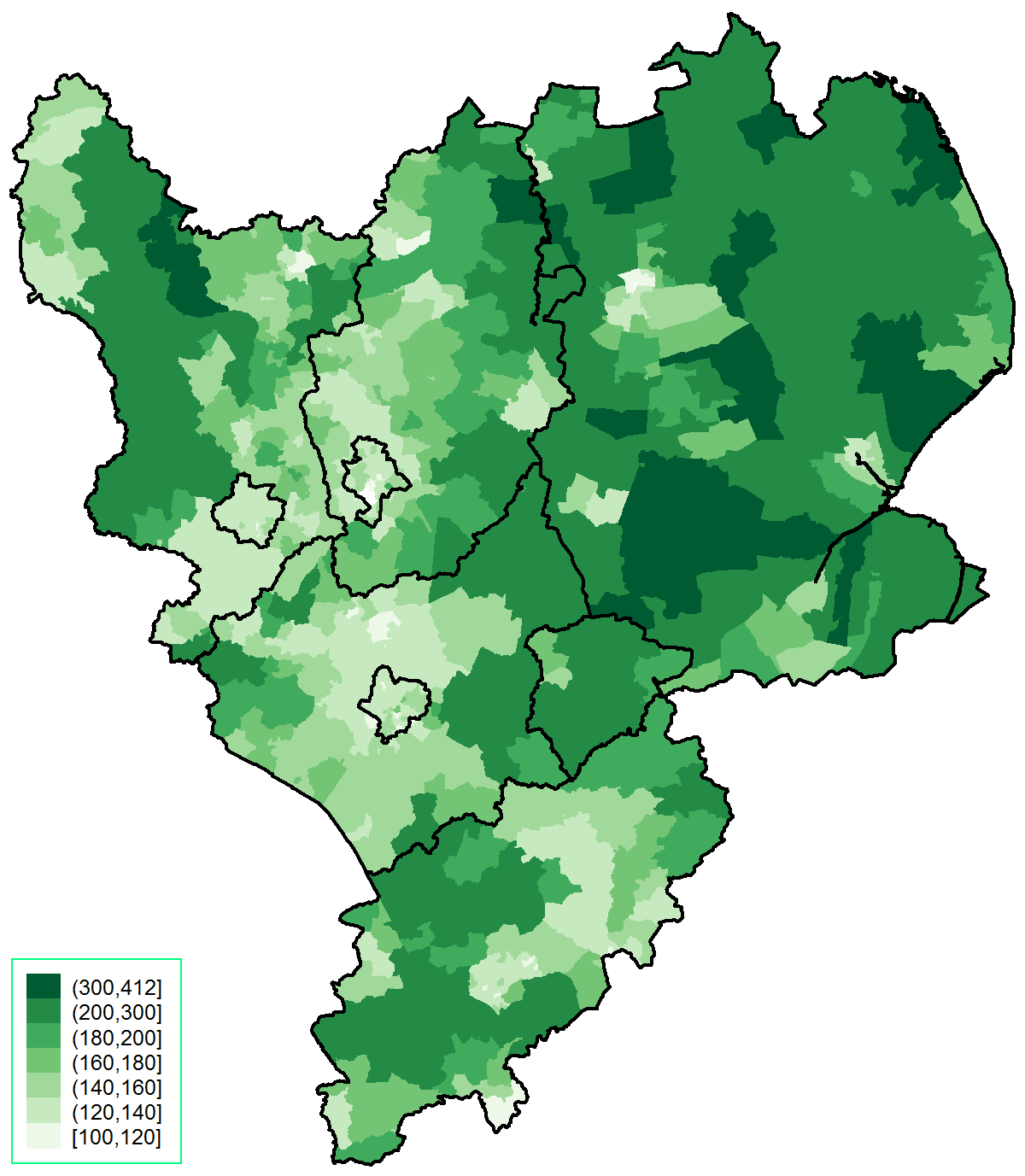


Figure B17: West Midlands


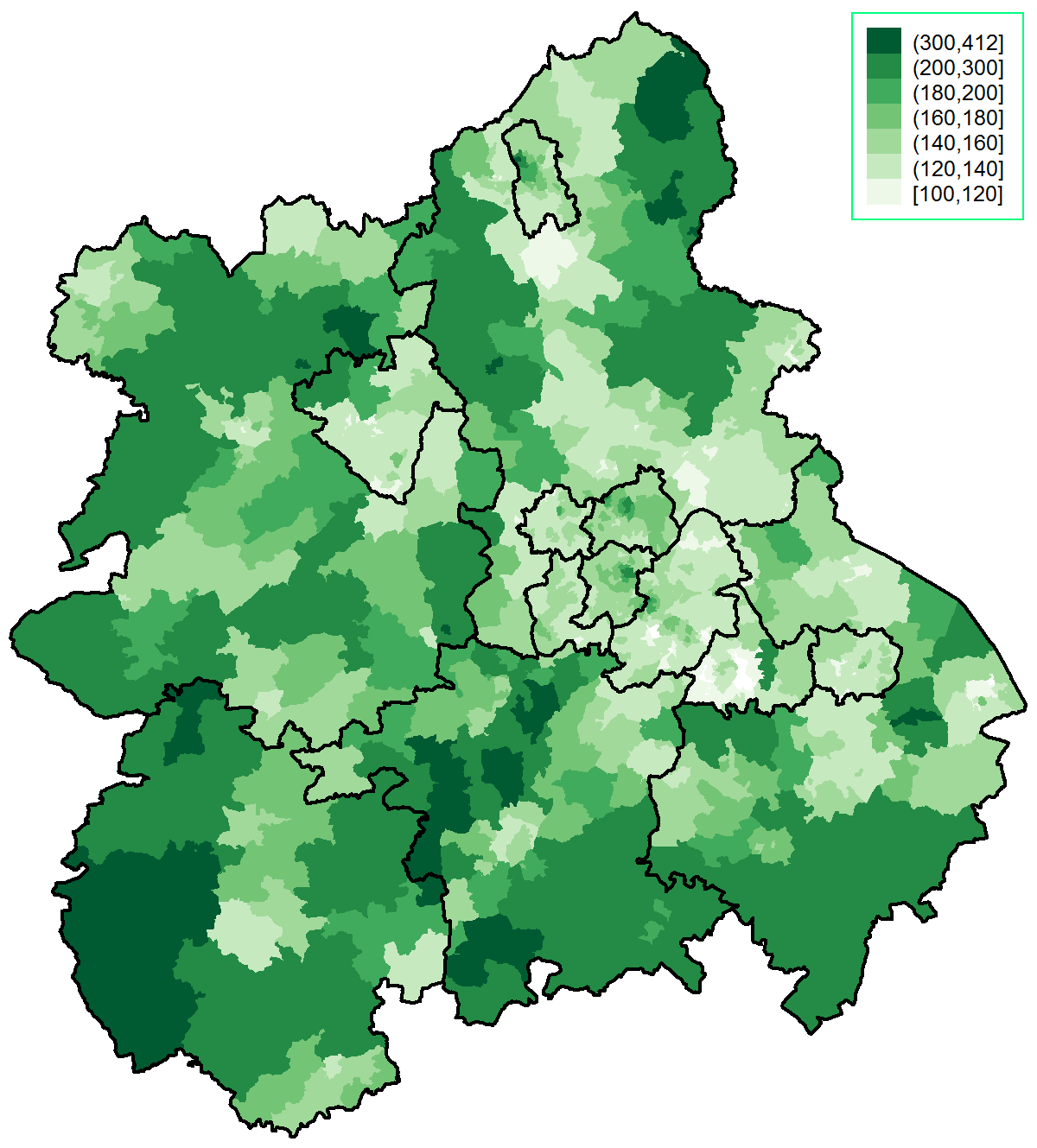


Figure B18: East of England


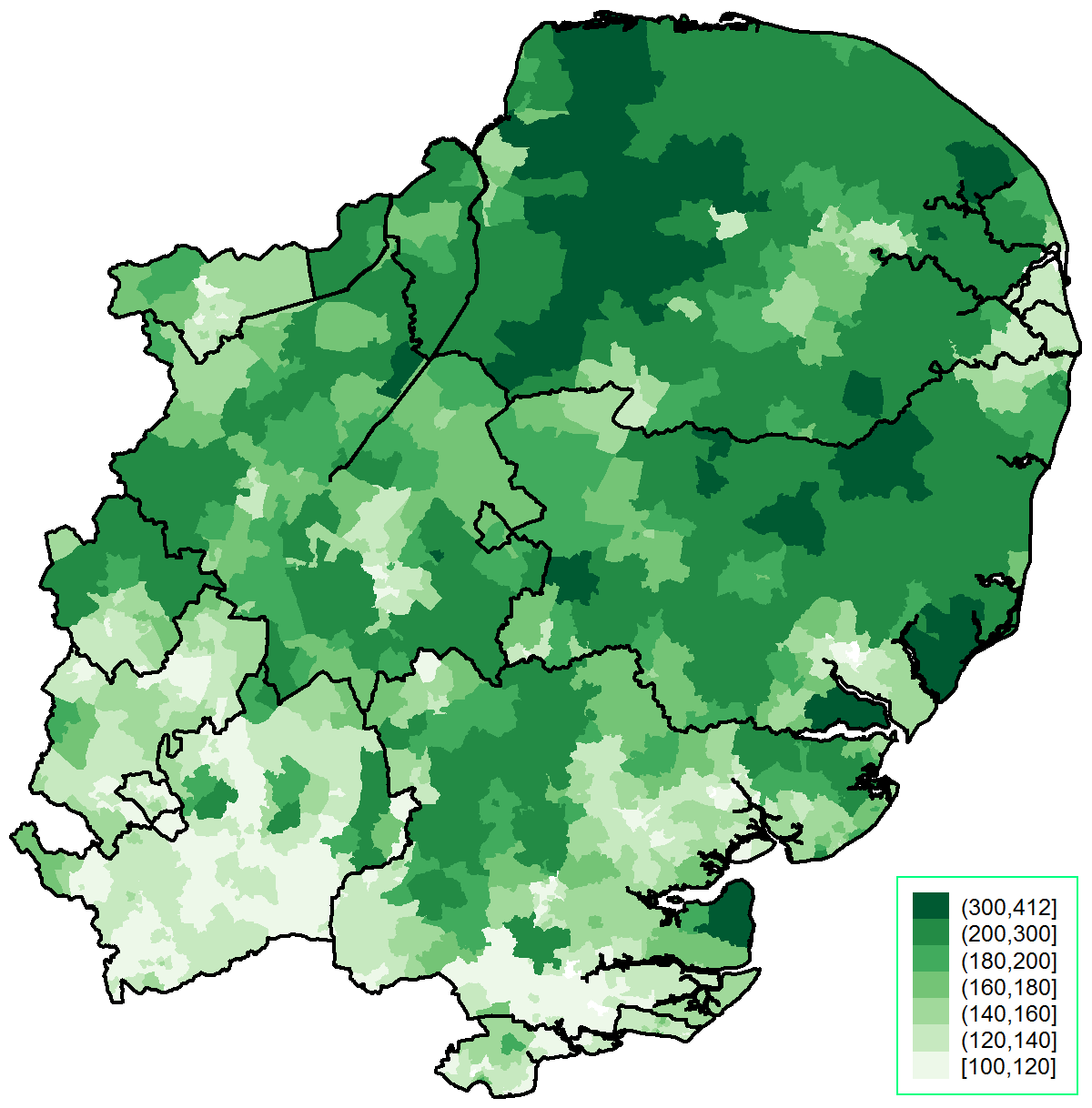


Figure B19: London


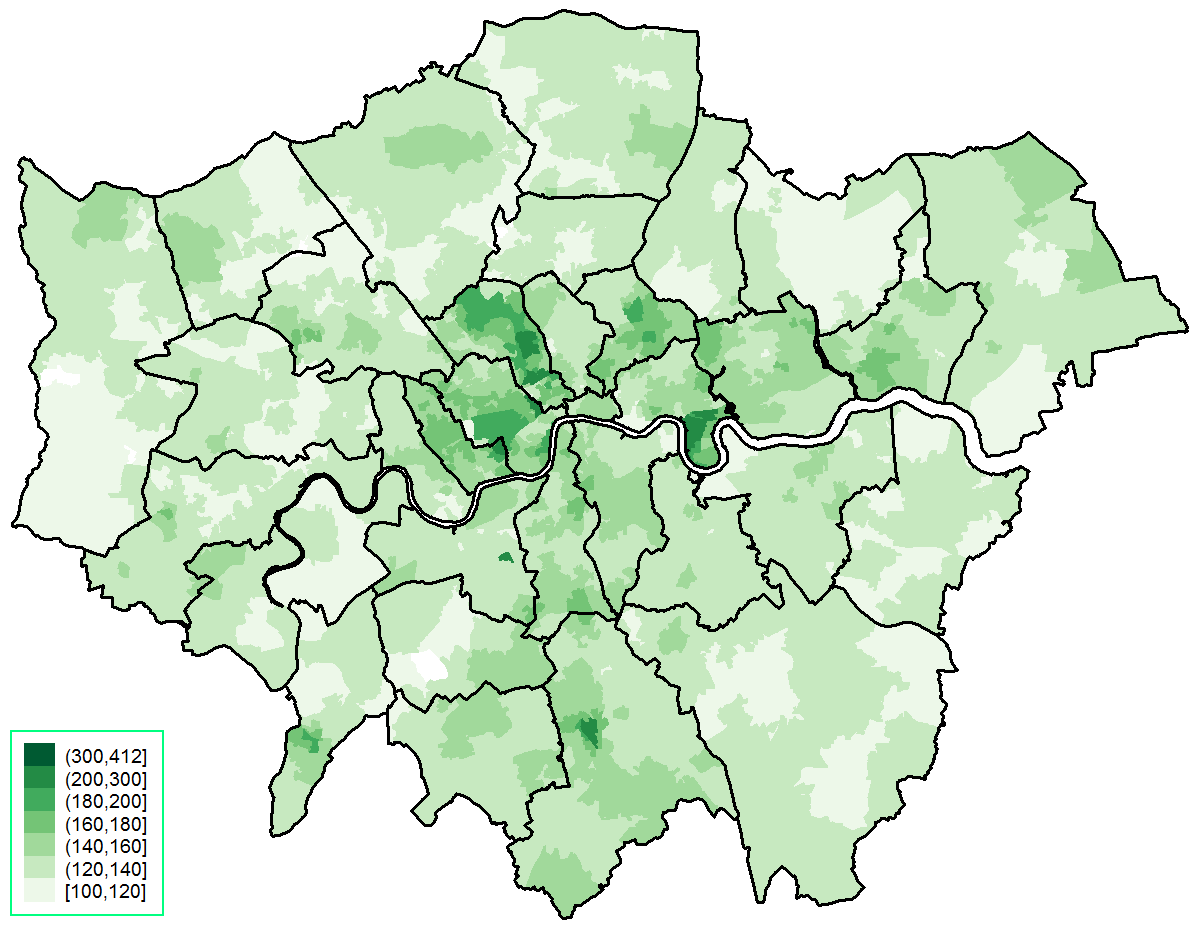


Figure B20: South East Coast


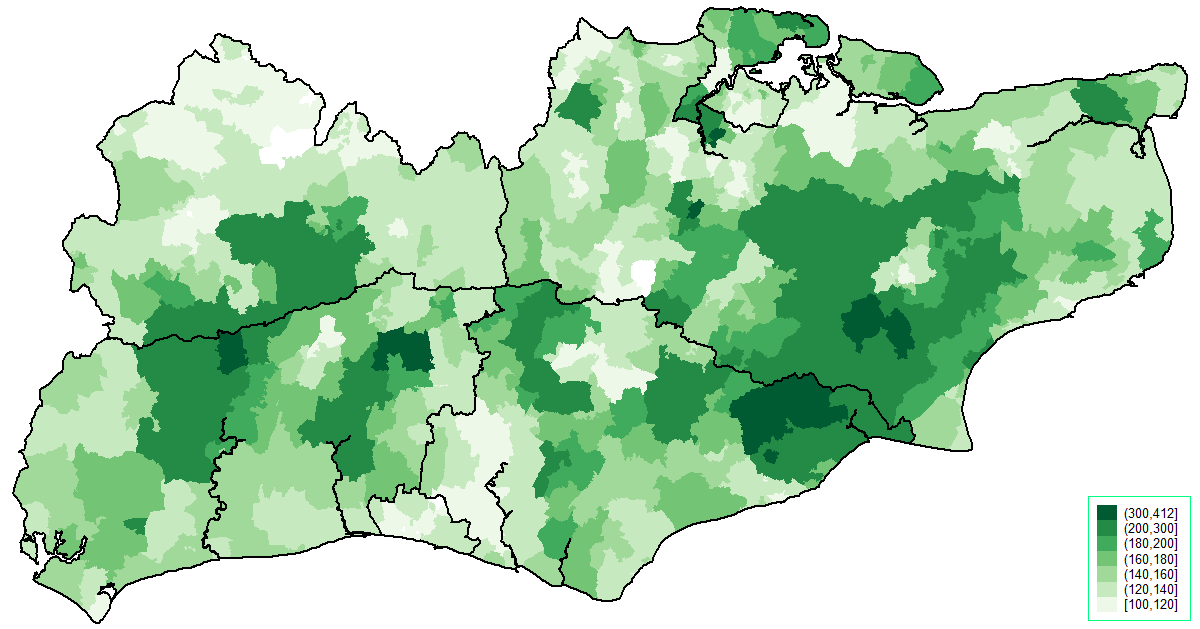


Figure B21: South Central


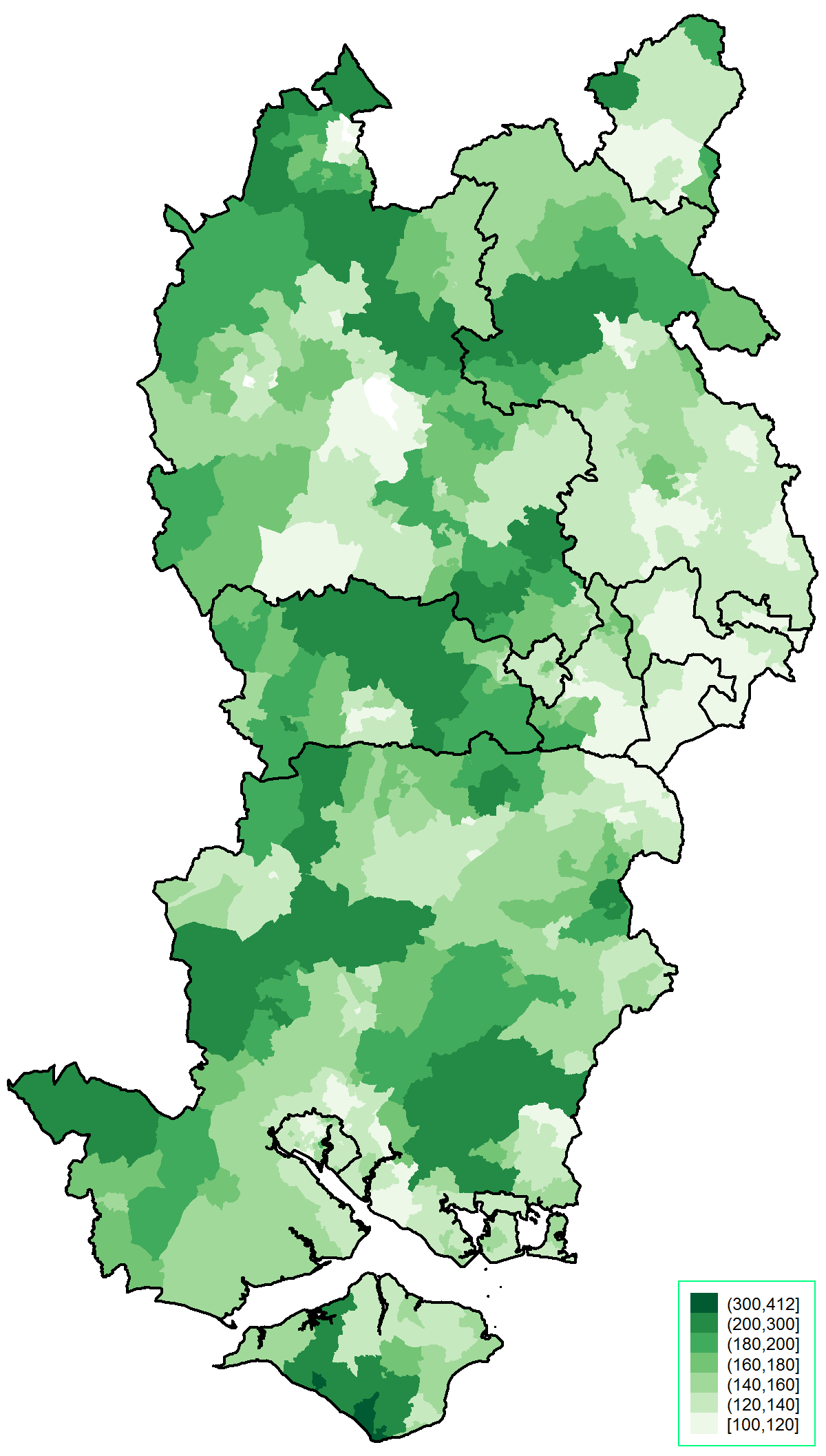


Figure B22: South West


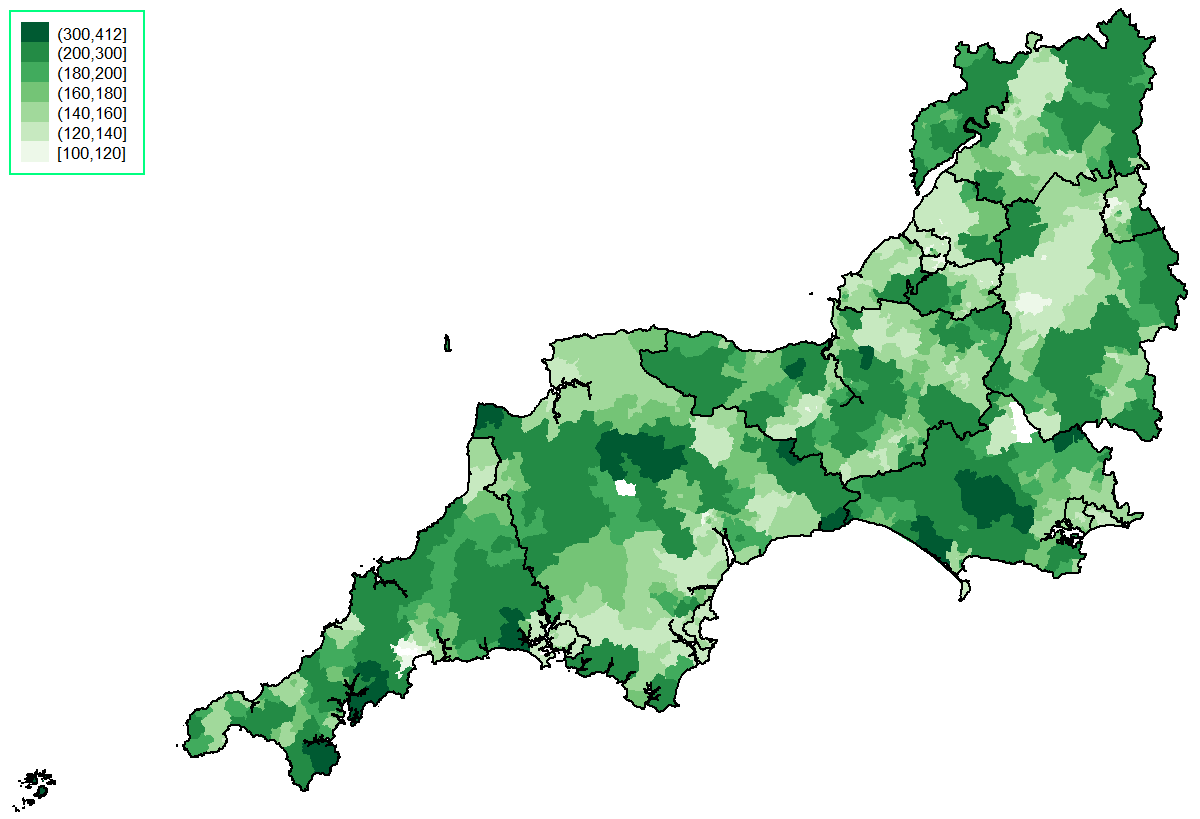


Figure B23: Greater Manchester (North West sub-region)


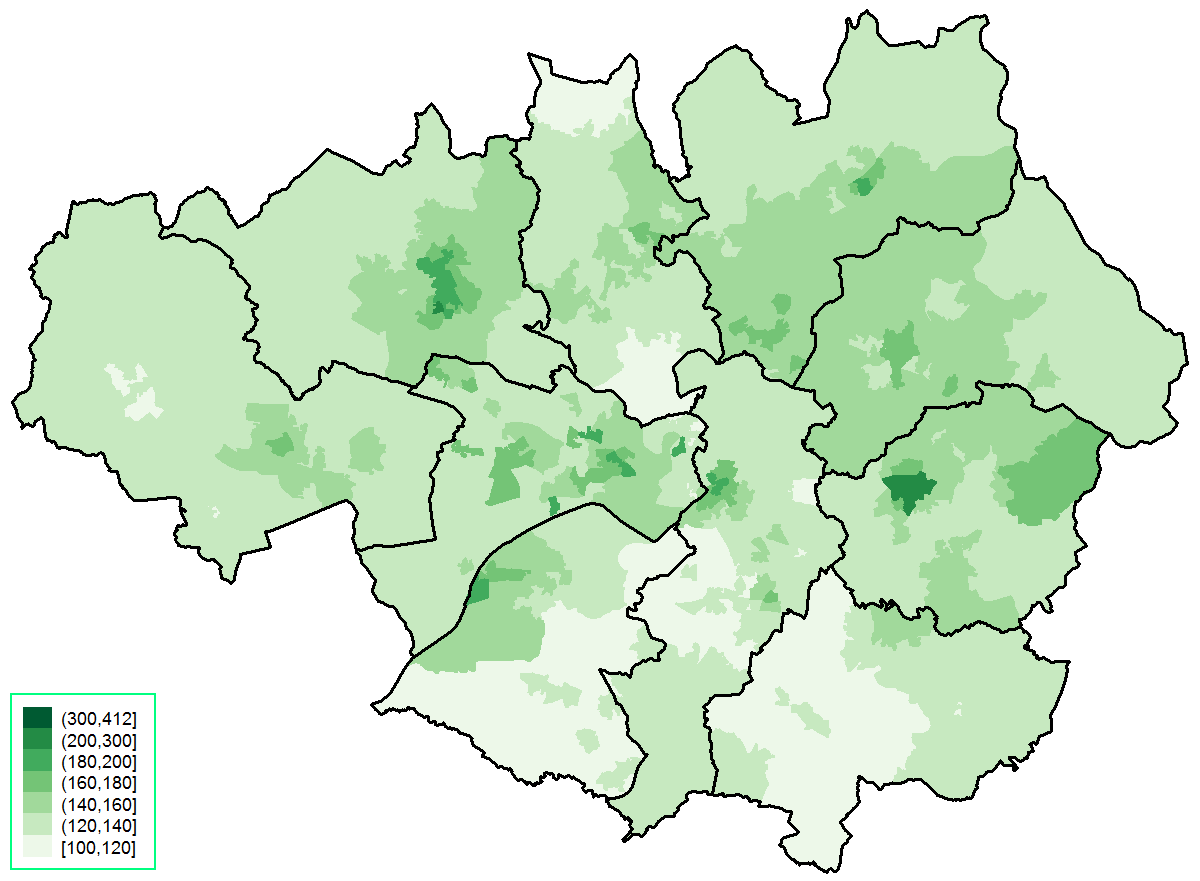


Figure B24: Birmingham (West Midlands sub-region)


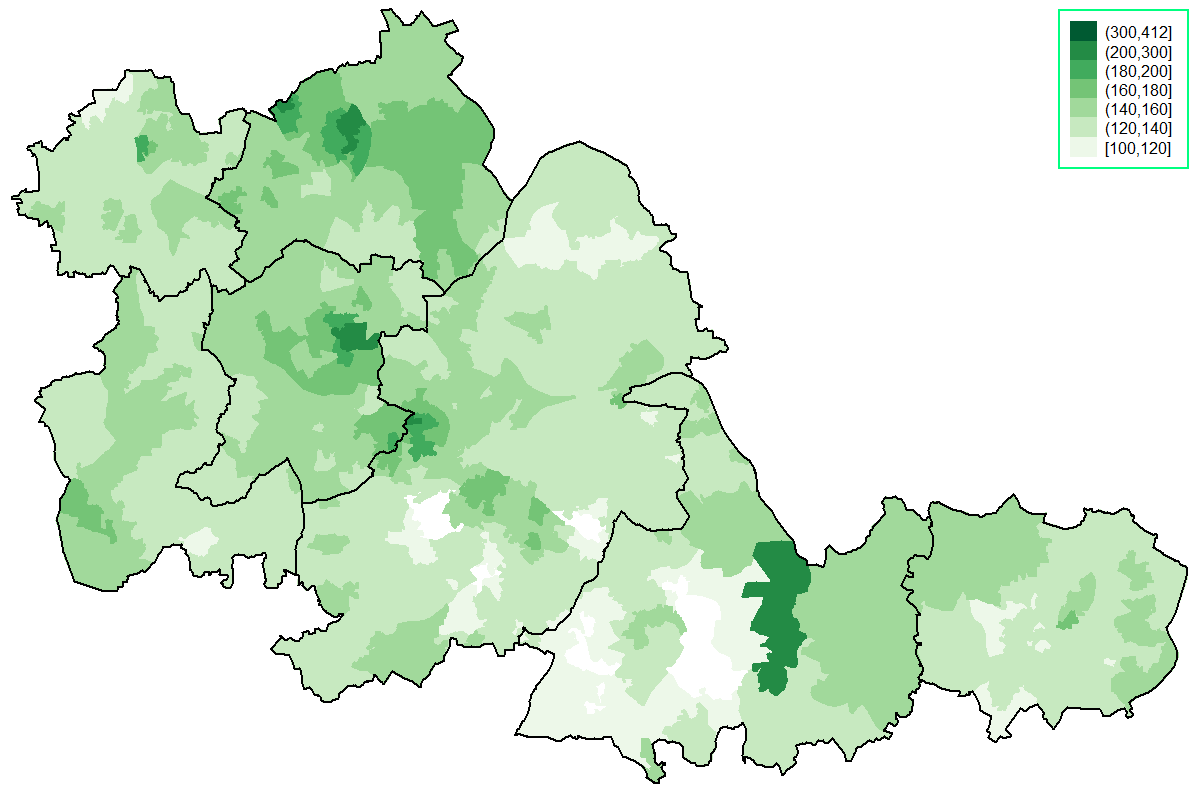

Supplement: Supplementary file 2 — Spatial maps by English region. (DOCX 3327 kb) [file 12916_2017_996_MOESM2_ESM.docx]
