# Supplementary material for: Chronic morbidity, deprivation and primary medical care spending in England in 2015-16: a cross-sectional spatial analysis
Source: BMC Med. 2018 Feb 14;16:19. doi: 10.1186/s12916-017-0996-0 (PMC5812046; doi:10.1186/s12916-017-0996-0)
Supplement: Supplementary file 3 — Scatter plots for deprivation vs. funding and morbidity vs. funding. (DOCX 325 kb) [file 12916_2017_996_MOESM3_ESM.docx]

# **Online appendix 3: Scatter plots for deprivation vs funding & morbidity vs funding**

Figure C1: Scatter plot of average primary care costs for 2015-16 by the morbidity ratio (top) and the 2015 Index of Multiple Deprivation (bottom), across all English Lower Super Output Areas (LSOAs)

| 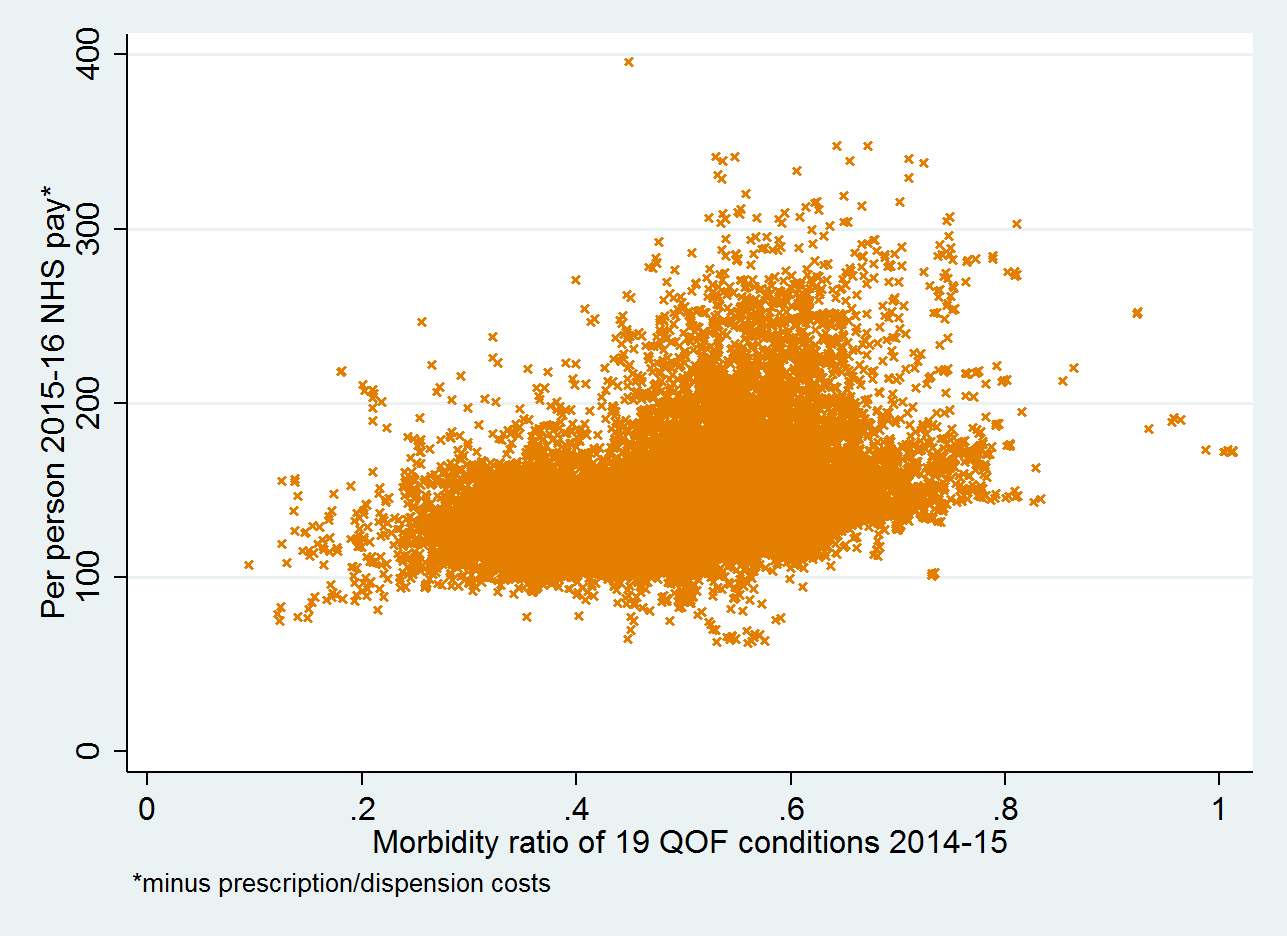 |
| --- |
| 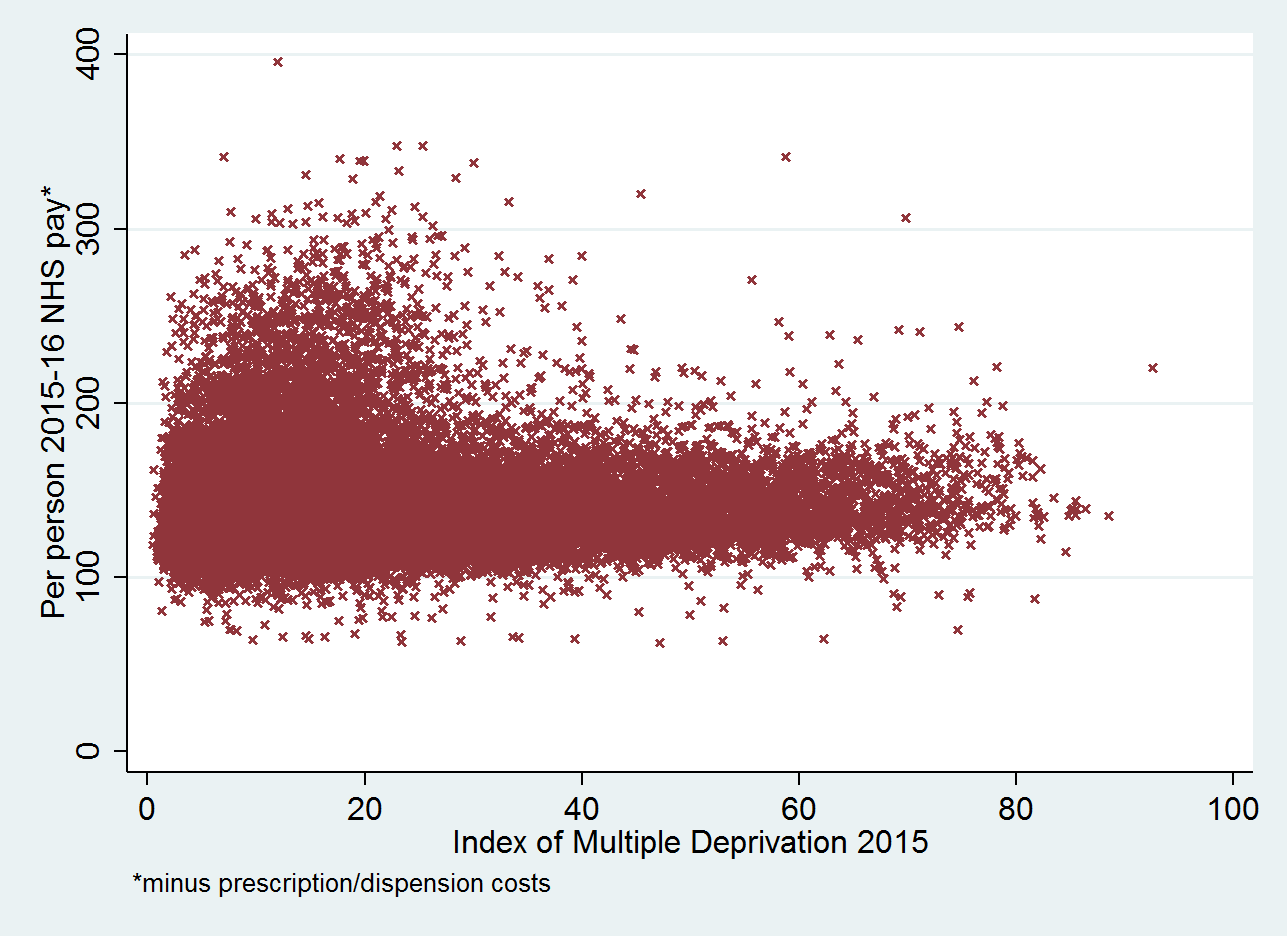 |

Figure C2: Scatter plot of population weighted average primary care costs for 2015-16 by the morbidity ratio (top) and the 2015 Index of Multiple Deprivation (bottom), across all English Clinical Commissioning Groups (CCGs)

| 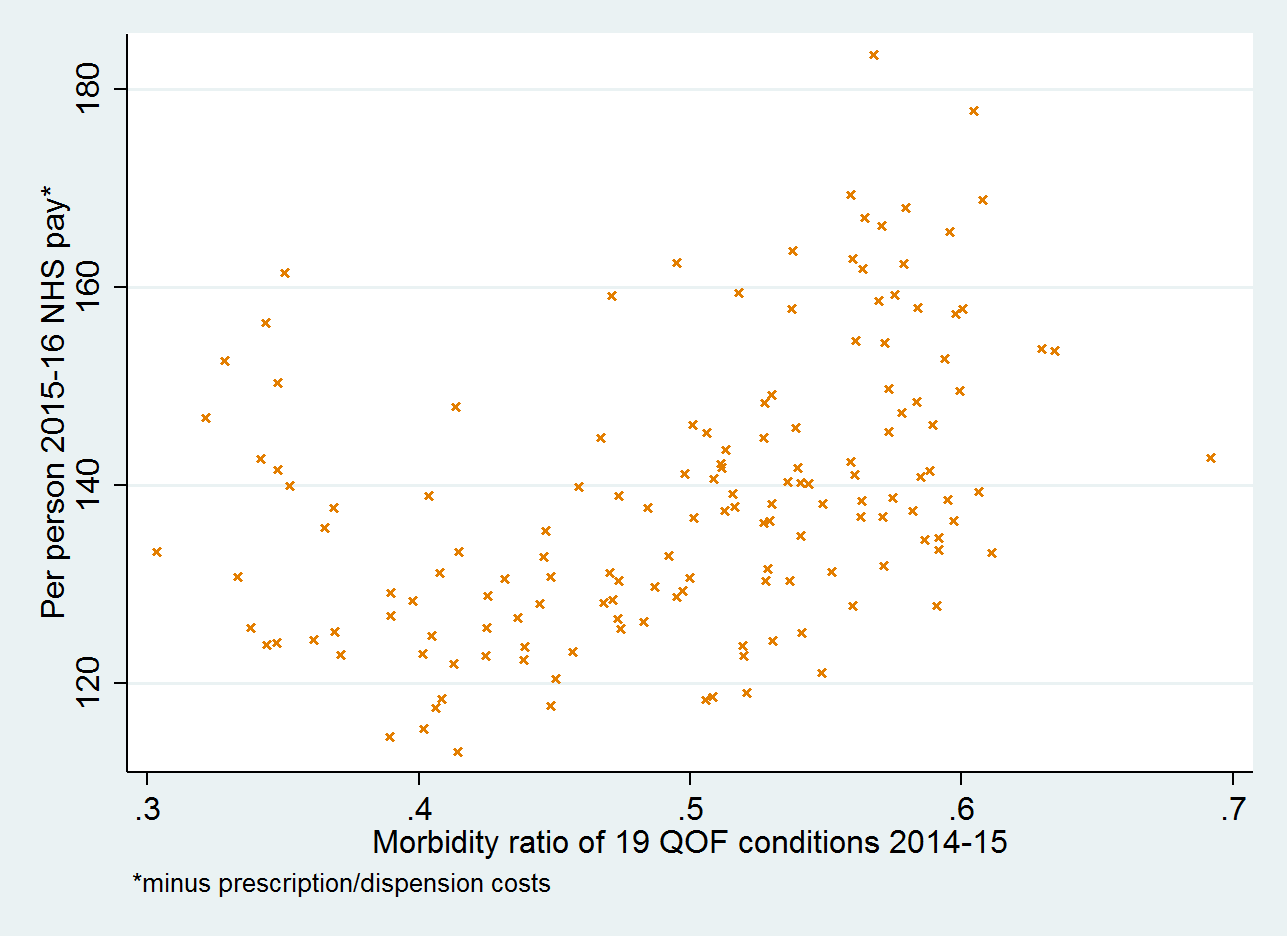 |
| --- |
| 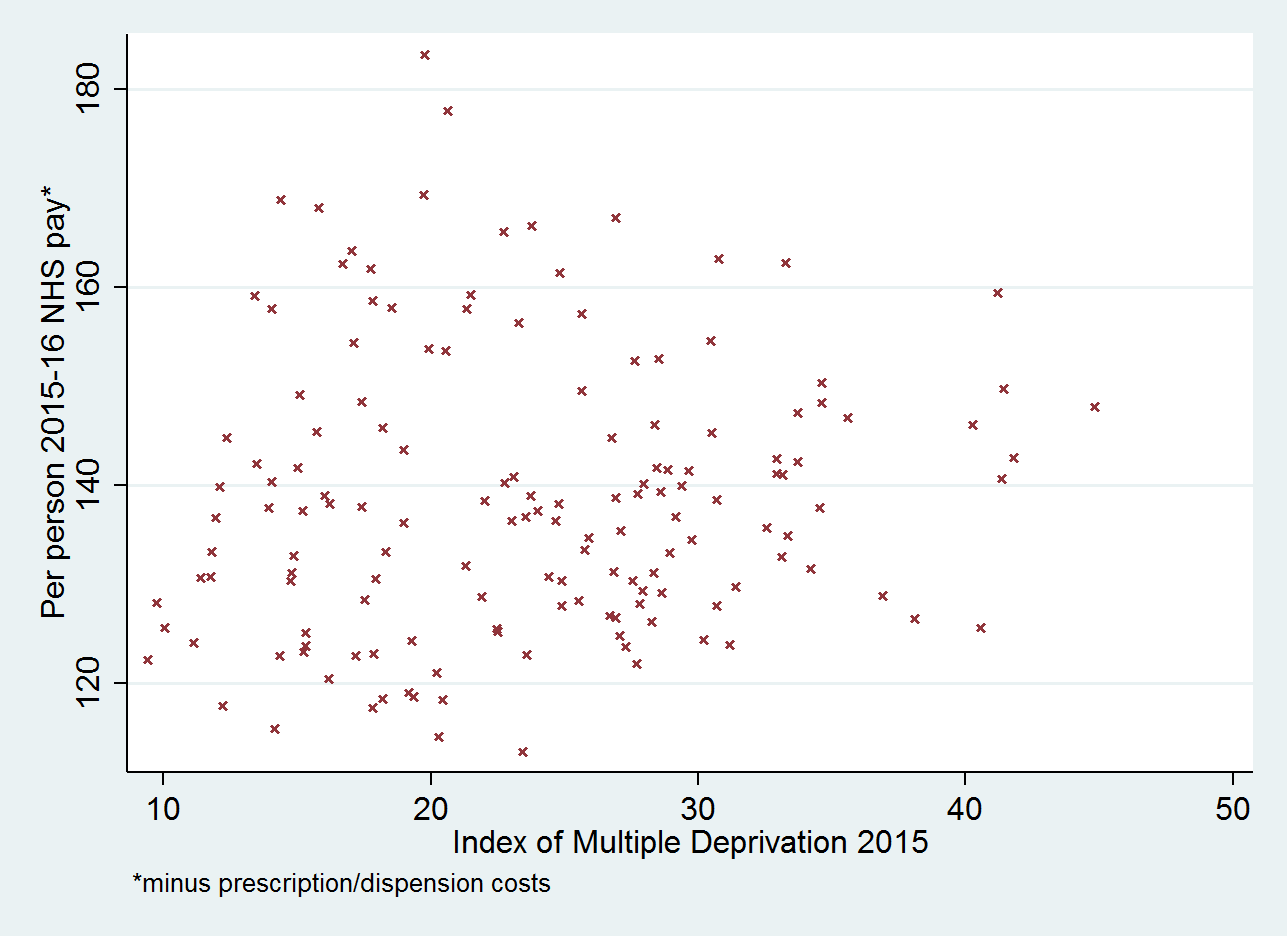 |
